# The Discriminative Efficacy of Retinal Characteristics on Two Traditional Chinese Syndromes in Association with Ischemic Stroke

**DOI:** 10.1155/2020/6051831

**Published:** 2020-03-26

**Authors:** Y. Y. Zhuo, J. M. Wu, L. Kuang, Y. M. Qu, B. Zee, J. Lee, Z. X. Yang

**Affiliations:** ^1^Department of Acupuncture and Moxibustion, Shenzhen Traditional Chinese Medicine Hospital, Shenzhen 518033, China; ^2^Department of Chinese Medicine, Affiliated Shenzhen Maternity and Child Healthcare Hospital, Southern Medical University, Shenzhen 518028, China; ^3^Department of Research and Education, Shenzhen University General Hospital, Shenzhen 518055, China; ^4^Division of Biostatistics, Jockey Club School of Public Health and Primary Care, Clinical Trials and Biostatistics Lab, CUHK Shenzhen Research Institute, Chinese University of Hong Kong, Hong Kong 999077, China

## Abstract

We aimed to investigate the efficacy of an objective method using AI-based retinal characteristic analysis to automatically differentiate between two traditional Chinese syndromes that are associated with ischemic stroke. Inpatient clinical and retinal data were retrospectively retrieved from the archive of our hospital. Patients diagnosed with cerebral infarction in the department of acupuncture and moxibustion between 2014 and 2018 were examined. Of these, the patients with Qi deficiency blood stasis syndrome (QDBS) and phlegm stasis in channels (PSIC) syndrome were selected. Those without retinal photos were excluded. To measure and analyze the patients' retinal vessel characteristics, we applied a patented AI-assisted automated retinal image analysis system developed by the Chinese University of Hong Kong. The demographic, clinical, and retinal information was compared between the QDBS and PSIC patients. The *t*-test and chi-squared test were used to analyze continuous data and categorical data, respectively. All the selected clinical information and retinal vessel measures were used to develop different discriminative models for QDBS and PSIC using logistic regression. Discriminative efficacy and model performances were evaluated by plotting a receiver operating characteristic curve. As compared to QDBS, the PSIC patients had a lower incidence of insomnia problems (46% versus 29% respectively, *p*=0.023) and a higher tortuosity index (0.45 ± 0.07 versus 0.47 ± 0.07, *p*=0.027). Moreover, the area under the curve of the logistic model showed that its discriminative efficacy based on both retinal and clinical characteristics was 86.7%, which was better than the model that employed retinal or clinical characteristics individually. Thus, the discriminative model using AI-assisted retinal characteristic analysis showed statistically significantly better performance in QDBS and PSIC syndrome differentiation among stroke patients. Therefore, we concluded that retinal characteristics added value to the clinical differentiation between QDBS and PSIC.

## 1. Introduction

Since 2012, stroke has been a leading cause of death and disability in China, and its incidence has been increasing at a rate of 8.7% per year [1]. The most common subtype of stroke in China is ischemic stroke, accounting for 43–79% of all stroke patients [2]. Traditional Chinese medicine (TCM) has been used in stroke treatment and recovery for thousands of years, and it is still commonly used in the clinical management of this condition [3]. One of the basic features of TCM for stroke is a treatment plan based on syndrome differentiation [4], that is, every TCM physician makes a diagnosis of stroke and individually prescribes medication based on each patient's syndrome differentiation. Therefore, the accuracy of the differentiation is the key to efficiently treating this disease.

According to the “Guidelines for the Diagnosis and Treatment of Common Diseases in the Traditional Chinese Medicine” [5], the syndromes (“ZHENG” in Chinese) of stroke are described in six aspects: wind pattern (Feng Zheng), heat pattern (Huo Re Zheng), phlegm pattern (Tan Zheng), blood stasis pattern (Xue Yu Zheng), qi deficiency pattern (Qi Xu Zheng), and yin deficiency pattern (Yin Xu Zheng). According to these syndromes, we arranged and combined them into seven syndrome types [6]. Of these, the Qi deficiency and blood stasis syndrome (QDBS) and the phlegm stasis in channels syndrome (PSIC) are most common syndrome types in ischemic stroke patients, occurring in about 53.6% of cases [7].

TCM treatment selection for stroke is based on the TCM syndrome types. Patients with QDBS are characterized by hemiplegia, weakness of limbs, numbness of the body, deviation of the tongue, swelling of hands and feet, pale complexion, shortness of breath, lack of strength, palpitation and spontaneous sweating, deviated and pale tongue with a thin white coating, and a fine gentle pulse, therefore improving Qi and promoting blood circulation is the first principle of treatment [7]. On the other hand, patients with PSIC are characterized by hemiplegia, deviation of the eye and mouth, stiff tongue, numbness of limbs, rapid arrest of hands and feet, dizziness, yellow sticky or greasy tongue coating, and a thready and slippery pulse; therefore, the treatment plan is focused on eliminating phlegm and freeing the channels [7].

An essential component of TCM diagnosis is an overall observation of human symptoms, which is defined as the TCM syndrome. However, due to the lack of standard and objective evaluation criteria, such diagnoses, can be influenced by the doctors' personal experience, which puts the repeatability and reliability of the diagnosis in question [8]. In lieu of this disadvantage, modern artificial intelligence and image analysis technology could build a possible link between biological measurements and clinical outcomes. AI technology may offer a new objective diagnostic method for TCM syndrome diagnosis, which may help to improve diagnostic accuracy of a veteran TCM practitioner.

One of the critical pathological changes of stroke is cerebral vascular change [9]. According to the current knowledge of embryogenesis and histology, the retinal vessel circulation system and cerebral vascular system share the same origin [10], and it has been demonstrated that the retinal vascular system is similar in function and morphology to the cerebral vascular system [10]. One of the hypotheses is that the alteration of the retinal image can reflect cerebral vascular changes, and this can be used as risk predictor for ischemic stroke [11]. Previous studies have shown that a number of retinal characteristics were significantly associated with stroke [12–16]. Furthermore, the retina is the only organ in the body whose vascular system can be observed noninvasively; therefore, the characteristics of retinal vasculature are considered as potential tools for stroke risk assessment. Unfortunately, to date, there has been no systematic investigation about the differences in retinal vessels between the various TCM syndrome types of stroke patients. In this study, we have explored the diagnosis of stroke syndromes in the context of TCM based on retinal images.

## 2. Materials and Methods

### 2.1. Ethical Statement

This study was approved by the Ethics Committee of the Shenzhen Traditional Chinese Medicine Hospital (Approval Number: 2018–75) and was performed in accordance with the guidelines of the Declaration of Helsinki (1964). All patients provided written informed consent for their participation in the study.

### 2.2. Study Design

In the case-control study, a total of 328 ischemic stroke patients from the Shenzhen Traditional Chinese Medicine Hospital were included. Patients were divided into 2 groups according to their TCM syndrome types: the QDBS group and the PSIC group. The patients' demographic and clinical data, including age, sex, medical history, physical examination, laboratory test, and electrocardiography results, were collected by trained doctors.

### 2.3. Patient Selection

**Figure 1:**
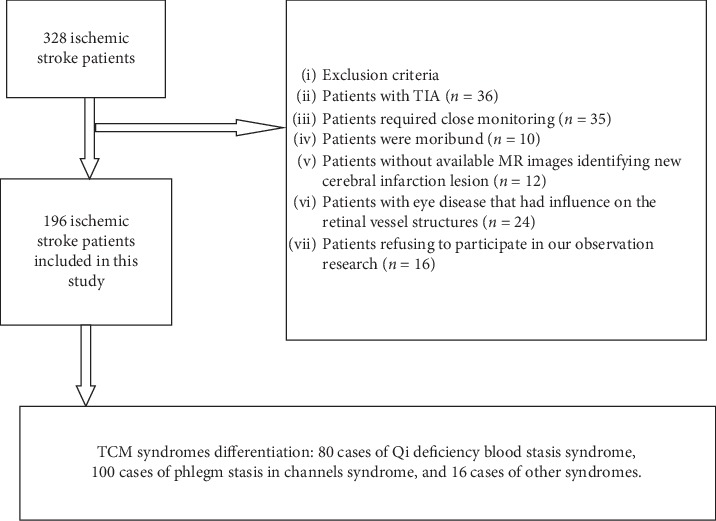
Flow chart of enrolment of the study population. MR, magnetic resonance; TIA, transient ischemic attack.

The inclusion criteria were as follows: the subject was in the recovery stage of ischemic stroke, aged between 30–80 and was adequately able to maintain his/her posture while sitting for the duration of the retinal photography procedure. If the subjects were found to be suffering from any of the following conditions, they were excluded from the study: clinically unstable and requiring close monitoring, moribund, had an eye disease that severely affected retinal vessel structures, or was physically or subjectively unable to comply with magnetic resonance (MR) examination. In addition, if the patient was suspected to have cerebral diseases or conditions that may potentially alter retinal vessel morphology, he or she was excluded as well. Eventually, 196 of the 328 patients with ischemic stroke were included in our study (Figure 1).

### 2.4. Data Collection

Risk factors related to cerebral infarction, such as hypertension, diabetes, dyslipidemia, high homocysteine, coronary heart disease, atrial fibrillation, smoking history, drinking history, and sleep disorders, were collected in this study. Hypertension was defined as a systolic blood pressure greater than 140 mmHg and a diastolic blood pressure above 90 mmHg or the use of antihypertensive medication for up to 2 weeks prior to the start of the study. According to diagnostic criteria from the National Diabetes Data Group, diabetes mellitus was defined as a fasting serum glucose level of more than 6.99 mmol/L, a nonfasting value of more than 11.1 mmol/L, or a history of treatment for diabetes [17]. Based on the National Cholesterol Education Program guidelines, dyslipidemia was classified as desirable (serum cholesterol level: <5.17 mmol/L), borderline-high (serum cholesterol level: 5.17–6.21 mmol/L), or history of administration of lipid-lowering drugs [18, 19]. Smoking and drinking status were evaluated by designating ex-smokers, current smokers, or nonsmokers and ex-drinkers, current drinkers, or nondrinkers, respectively. Physical activity and mental status were also investigated by assessing if the patients exercised regularly, felt despair, had a poor appetite, and slept enough.

Retinal images were taken on the 2^nd^ day of hospital admission using the SmartScope Ey4 Camera (Optomed, Finland). To ensure the compatibility of the parameters, all the retinal images were scaled to 1365^*∗*^1024 pixels and saved in the jpg format. A patented, fully automatic retinal image analysis was applied to measure the retinal vessel characteristics, including vessel diameter, vessel branching angle and bifurcation measures, vessel tortuosity, and fractal dimensions [12, 13, 20–24].

### 2.5. Statistical Analysis

We reported data as mean and standard deviation (mean ± SD) for continuous variables and as proportions for categorical variables. To analyze the manually measured clinical and retinal characteristics, we used 2 sample independent *t*-tests to compare the continuous data and chi-squared tests to compare the categorical data. A *p* value of <0.05 was considered statistically significant. We used nonparametric test (Mann-Whitney test) if the normality test showed rejection of normality assumption. For categorical data, exact Fisher's test was used if the expected count was less than five. A logistic regression model was used to build classification models. The steps used to establish these models were drawn from the method proposed by Hosmer and Lemeshow [25] for selecting independent variables that result in the best model. The classification accuracy and the area under the curve (AUC) of the receiver operating characteristic (ROC) were measured. All the data were analyzed using the Statistical Package for Social Science software (SPSS version 22.0, IBM Corp., Armonk, New York, USA).

## 3. Results

The parameters of sex, insomnia status, and tortuosity of retinal vessels were significantly different between the two TCM syndrome groups. Compared to the QDBS group, patients in the PSIC group had a significantly higher proportion of females (65% versus 81%, respectively, *p*=0.019), fewer insomnia problem (46% versus 29%, respectively, *p*=0.023), and a higher tortuosity index syndrome (0.45 ± 0.07 versus 0.47 ± 0.07, *p*=0.039). The drinking status presented a certain difference between the two syndromes with borderline significant difference (*p*=0.051). On the other hand, no statistically difference was found between the two groups in terms of the other clinical risk factors such as hypertension, diabetes, coronary heart disease (CHD), lipid level, or homocysteine level (Table 1).

**Figure 2:**
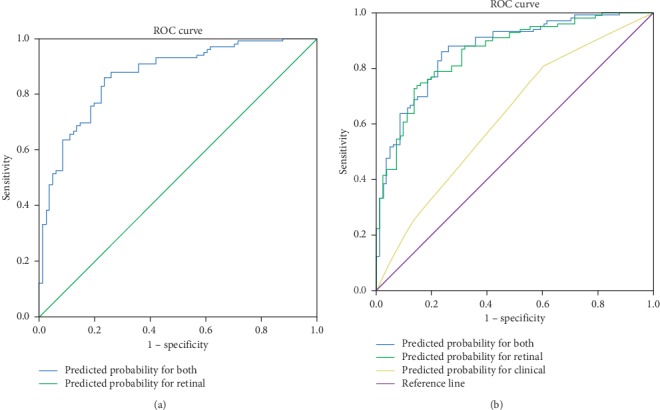
ROC curve for model classification. (a) ROC curve for the model based on clinical and retinal characteristics. (b) ROC curve for the model based on different sets of characteristics.

The stepwise logistic regression method was used to build classification models that included only clinical variables, only retinal variables, and combination of both clinical and retinal variables together. For the clinical model, the insomnia status (*p*=0.017) and drinking history (*p*=0.039) were significant. The percentages of correct classification for PSIC and QDBS were 39.5% and 80.8%, respectively. The model that used retinal characteristic variables alone included AVR (*p*=0.053), bifurcation coefficient of venule (*p*=0.003), hemorrhage (*p*=0.025), and arterial occlusion (*p*=0.033), and a composite score of retinal interactions, showed that the percentages of correct classification were 70.4% and 80.8% for PSIC and QDBS, respectively. A final logistic regression model combined both clinical and retinal variables included insomnia status (*p*=0.020), AVR (*p*=0.099), BCV (*p*=0.006), hemorrhage (*p*=0.019), arterial occlusion (*p*=0.030), and a composite score of interactions (*p*=0.001). The percentages of correct classification were 76.5% and 84.8% in PSIC and QDBS, respectively. The odds ratios (OR) and the corresponding 95% confidence intervals (95% CI) of each of the significant variables are shown in Table 2. The area under the ROC curve was 0.867 (Figure 2(a); 95% CI: 0.81–0.92).

The AUCs were 0.624 (95% CI: 0.542–0.706, *p*=0.004) for model 1 with clinical variables alone, 0.857 (95% CI: 0.804–0.911, *p* < 0.001) for model 2 with retinal variables alone, and 0.867 (95% CI: 0.815–0.920, *p* < 0.001) for model 3 with both clinical and retinal characteristics. Compared with model 1, the AUCs of model 2 and model 3 have statistically significant increased (*p* < 0.001, *Z* = 4.85 and *Z* = 6.15, respectively). The increase in AUC from model 2 to model 3 was not statistically significant (Figure 2(b)).

## 4. Discussion

In practice, the four clinical TCM diagnosing techniques, that is, observation, auscultation and olfaction, interrogation, and pulse feeling and palpation are combined to identify a specific disease. However, obtaining a correct diagnosis using these techniques is highly dependent on the domain knowledge of the TCM physicians. For instance, most of the diagnostic information is gathered by the naked eye and subjective feeling during assessment of each physician. Therefore, our proposed method of using an objective AI-based retinal characteristic analysis is considered a highly valuable approach in the clinical practice of traditional Chinese medicine.

At present, there is a vast difference between the procedure of standardization of the TCM syndrome classification of syndrome type and the method of statistical processing. The clinical methods suitable for standardizing TCM syndrome classification include cluster analysis, factor analysis, principal component analysis, artificial neural networks, regression analysis, and discriminant analysis [26–28]. Collecting accurate representative clinical characteristics and using a correct method of data analysis can ensure a very high reliability of TCM syndrome classification. Our research was based on the clinical history data, so we could establish an optimal discriminant function and regression equation, in effect, achieving a mathematical summary of the data.

In recent years, there have been many clinical studies on the correlation between objective indicators and the TCM syndrome types of ischemic stroke, of which the indicators in question have been biochemical indicators, imaging indicators, and scale evaluations [29–31]. In 2013, a prospective cohort study of the Asian population in Malaysia proposed that the addition of retinopathy tests can improve the physician's ability to predict a stroke [32]. Therefore, we included retinal characteristics and clinical risk factors to classify the syndrome types of ischemic stroke in our study.

Insomnia and alcohol consumption are related to the occurrence ischemic stroke and are considered to be its risk factors. Insomnia is a common symptom that is associated with increased risk of mortality in first-time stroke patients [33]. A high frequency of drinking before the stroke is related to an all-cause mortality in patients with ischemic stroke [34]. In our study, we focused on the clinical risks of ischemic stroke and demonstrated that a history of insomnia or drinking could indicate the presence the two TCM syndromes, QDBS and PSIC. Shi [35] found that Qi deficiency was diagnosed in 87.43% of patients with insomnia and spiritlessness. This finding coincides with our result that patients of ischemic stroke with QDBS syndrome were more likely to manifest insomnia. This can be explained from the TCM theory that fatigue due to lack of sleep is the key component of Qi deficiency and eventually results in blood stasis. According to TCM somatology, alcohol promotes sweet and bitter tastes and excessive drinking can lead to a hot and damp manifestation in the body, eventually causing the production of phlegm. Vascular damage appears to become worse with the accumulation of phlegm, which results in ischemic stroke. Furthermore, in our study, patients with PSIC syndrome were found to have a higher percentage of drinking history, which is concurrent with the study by Zhu et al. [36], which stated that the risk factors of a phlegm-wetness body type were caused by alcohol consumption.

A cross-sectional study based on two community populations in southeastern United States explored the positive relationship between fundic vascular anomalies and cerebral infarction and confirmed it via MRI [37]. This study found that there was a correlation between cerebral infarction and fundic vascular anomalies, evidenced by arteriovenous local stenosis, local vasoconstriction, punctate hemorrhage, soft exudates, and microaneurysms. The ARIC [38] study in 2010 found that the decrease of the central retinal arteriole equivalent (CRAE), the increase of the central retinal vein equivalent (CRVE), the stenosis of small arteries, and the cross-pressure of arteries and veins were associated with lacunar infarction. A meta-analysis showed that thinning of the retinal arterioles, arteriovenous cross-pressure, hemorrhage, microaneurysms, and a reduction of fractal dimension were also associated with stroke [39]. In our study, we found that vascular distortion and soft exudates were consistent with the risk of cerebral infarction, apart from the retinal characteristics. Our study also found that some retinal characteristics could be used to classify these 2 TCM syndromes. Characteristics of vascular morphology and integrity, such as AVR, BCV, hemorrhage, arterial occlusion, were more significantly altered in the QDBS group.

In this study, we differentiated between the two TCM syndromes of ischemia stroke using different retinal characteristics. A model that facilitated this differentiation model displayed higher diagnostic efficacy based on both retinal vessel characteristics and clinical variables, rather than being dependent only on clinical variables. In fact, the model based on retinal variables alone is almost as good as the combined model with both clinical and retinal variables. Therefore, the retinal vessel features that were obtained by us could be used for interpretation and guidance of stroke syndromes in TCM clinical practice.

Several potential study limitations should be considered. First, all the participants included in this study were recruited in the same hospital. A multicenter clinical study should be designed to fully investigate our results. Second, in addition to QDBS and PSIC, the other five TCM syndrome types should be investigated in future studies. Third, due to the small sample size of our study, a test data was not conducted in this study. Future studies on this topic should include larger sample sizes. However, we have carried out a leave-one-out cross-validation for the final model. The percentages of correct classification for PSIC and QDBS were 77.8% and 74.7%, respectively.

## 5. Conclusions

In this study, we developed a logistic regression diagnosis model by combining clinical variables and retinal characteristics. We collected information on clinical variables and used the features extracted automatically from retinal images to create an objective method of diagnosis of ischemic stroke. This model is effective for distinguishing between the two TCM syndromes of ischemic stroke. Therefore, we concluded that retinal characteristics are useful for clinically differentiating between QDBS and PSIC.

## Figures and Tables

**Table 1:** Clinical and retinal characteristics of the two TCM syndrome groups.

Characteristics	QDBS group (*n* = 81)	PSIC group (*n* = 99)	*p* value
Clinical characteristics			
Age (years)	62.185 ± 11.617	61.283 ± 12.742	0.820^*∗*^
BMI (kg/m^2^)	23.405 ± 3.765	23.21 ± 2.663	0.627^*∗*^
Sex			0.019
Male	28 (34.6%)	19 (19.2%)	
Female	53 (65.4%)	80 (80.8%)	
Drinking			0.051
No	70 (86.4%)	74 (74.7%)	
Yes	11 (13.6%)	25 (25.3%)	
Insomnia			0.023^*∗*^
No	44 (54.3%)	70 (70.7%)	
Yes	37 (45.7%)	29 (29.3%)	
Hypertension			0.844
No	19 (23.5%)	22 (22.2%)	
Yes	62 (76.5%)	77 (77.8%)	
Diabetes			0.620
No	42 (51.9%)	55 (55.6%)	
Yes	39 (48.1%)	44 (44.4%)	
CHD			0.588
No	61 (75.3%)	71 (71.7%)	
Yes	20 (24.7%)	28 (28.3%)	
Abnormal lipid level			0.560
No	54 (66.7%)	70 (70.7%)	
Yes	27 (33.3%)	29 (29.3%)	
High homocysteine level			0.340
No	74 (91.4%)	86 (86.9%)	
Yes	7 (8.6%)	13 (13.1%)	
Retinal characteristics			
CRAE	12.378 ± 1.195	12.451 ± 1.209	0.826^*∗*^
CRVE	18.897 ± 1.207	19.058 ± 1.229	0.364^*∗*^
AVR	0.650 ± 0.024	0.644 ± 0.023	0.117^*∗*^
Vasym	0.791 ± 0.013	0.789 ± 0.015	0.322
Aasym	0.8240 ± 0.014	0.8248 ± 0.014	0.704
Vangle	74.570 ± 2.414	74.450 ± 2.141	0.388^*∗*^
Aangle	70.150 ± 2.271	69.869 ± 2.066	0.492^*∗*^
BCV	1.352 ± 0.054	1.346 ± 0.052	0.510^*∗*^
BCA	1.743 ± 0.050	1.745 ± 0.046	0.589^*∗*^
Tortuosity	0.452 ± 0.067	0.474 ± 0.073	0.027^*∗*^
Nicking	0.236 ± 0.051	0.240 ± 0.043	0.539
Hemorrhage	0.330 ± 0.078	0.346 ± 0.089	0.218
Aocclusion	0.228 ± 0.087	0.216 ± 0.085	0.428^*∗*^
Exudates	0.346 ± 0.091	0.341 ± 0.088	0.686

^*∗*^The Mann–Whitney test was used due to skewness of the data; detail information is given in the supplementary. CRAE: central retinal artery equivalent; CRVE: central retinal vein equivalent; AVR: arterial-venous ratio; Aasym: asymmetry index of arterioles; Vasym: asymmetry index of venules; Vangle: bifurcation angles of venule; Aangle: bifurcation angles of arterioles; BCV: bifurcation coefficient of venule; BCA: bifurcation coefficient of arterioles; Nicking: the narrowing of the venule at the crossing point of arteriole. Aocclusion: arterial occlusion.

**Table 2:** Multivariable logistic regression for the two TCM syndromes of ischemic stroke.

Characteristics	OR	95% CI	*p* value
Insomnia	0.379	0.167–0.861	0.020
AVR	0.838	0.680–1.034	0.099
BCV	1.163	1.045–1.294	0.006
Hemorrhage	2.218	1.137–4.325	0.019
Aocclusion	0.545	0.314–0.944	0.030
Composite score	1.820	1.529–2.166	0.001

AVR: arterial-venous ratio; BCV: bifurcation coefficient of venules; Aocclusion: arterial occlusion.

## Data Availability

The EXCEL data used to support the findings of this study are available from the corresponding author upon request.

## Supplementary Materials

Supplementary Materials These are the retinal images of 196 patients recruited in the study. Supplementary for univariate analysis.
