# Supplementary material for: The Discriminative Efficacy of Retinal Characteristics on Two Traditional Chinese Syndromes in Association with Ischemic Stroke
Source: Evid Based Complement Alternat Med. 2020 Mar 26;2020:6051831. doi: 10.1155/2020/6051831 (PMC7136789; doi:10.1155/2020/6051831)
Supplement: Supplementary Materials — These are the retinal images of 196 patients recruited in the study. Supplementary for univariate analysis. [file 6051831.f1.zip › 6051831.f1/mat.6051831.v3 (1).pdf]

Supplementary for univariate analysis:

Part1. For continuous variables

a) Clinical characteristics

Tests of Normality

	Kolmogorov-Smirnov[a]			Shapiro-Wilk		
	Statistic	df	Sig.	Statistic	df	Sig.
age	.083	195	.002	.979	195	.005
BMI	.095	195	.000	.925	195	.000

a. Lilliefors Significance Correction

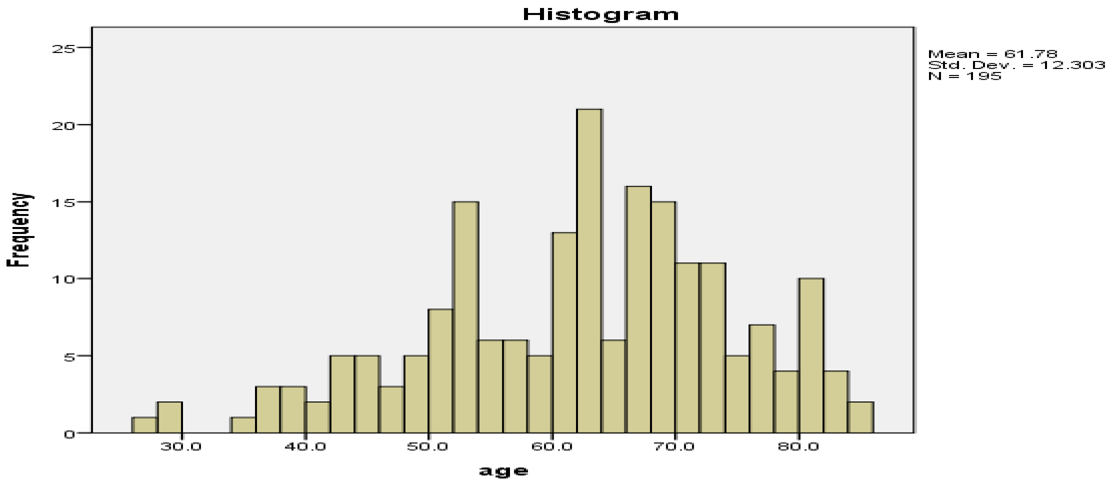

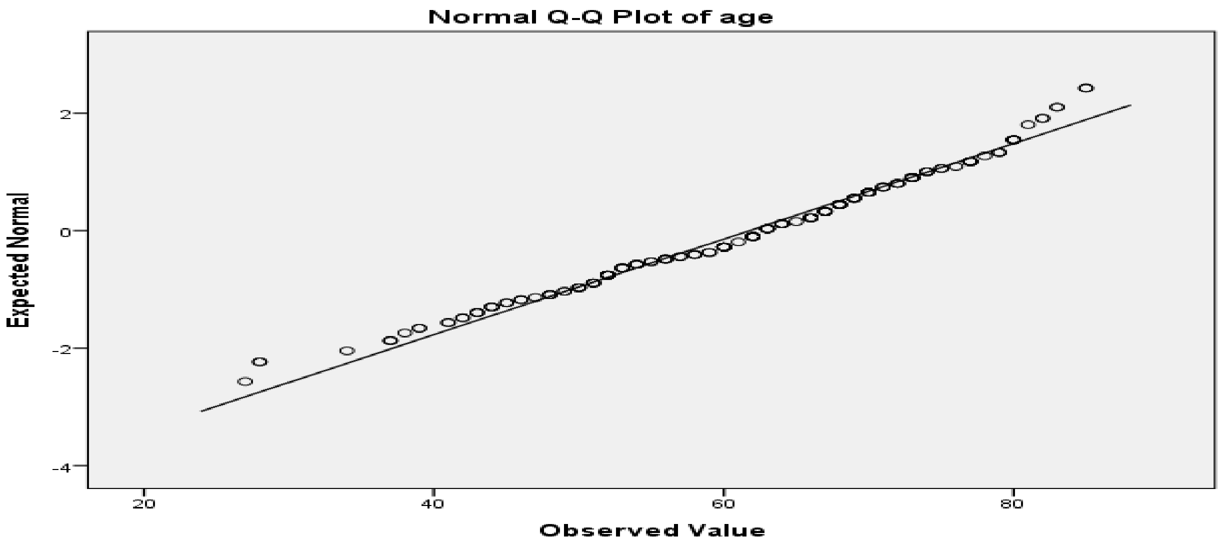

Comments: slightly skew to left. For safety we may use nonparametric test.

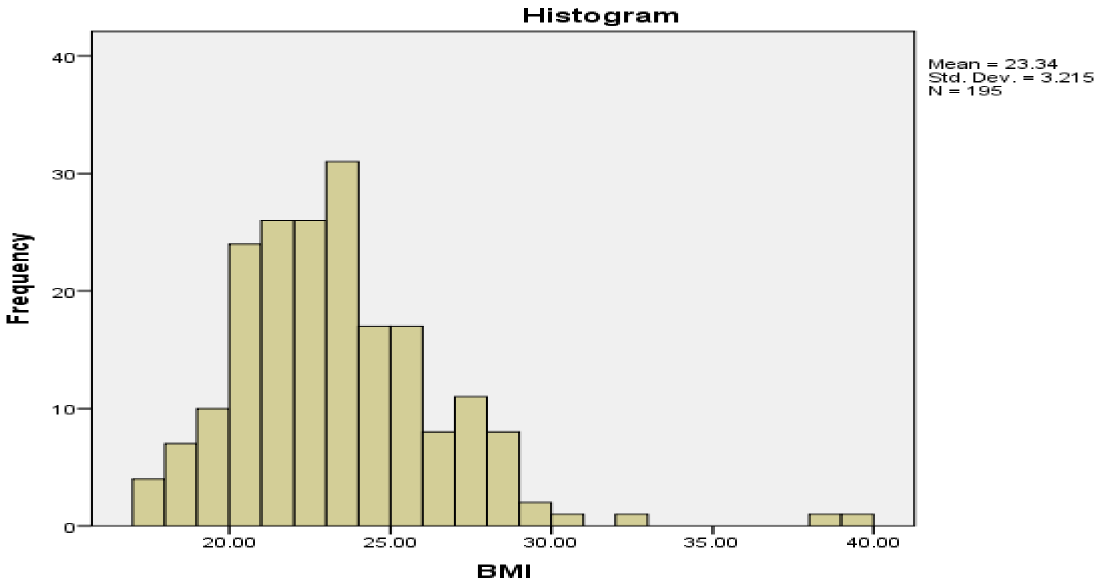

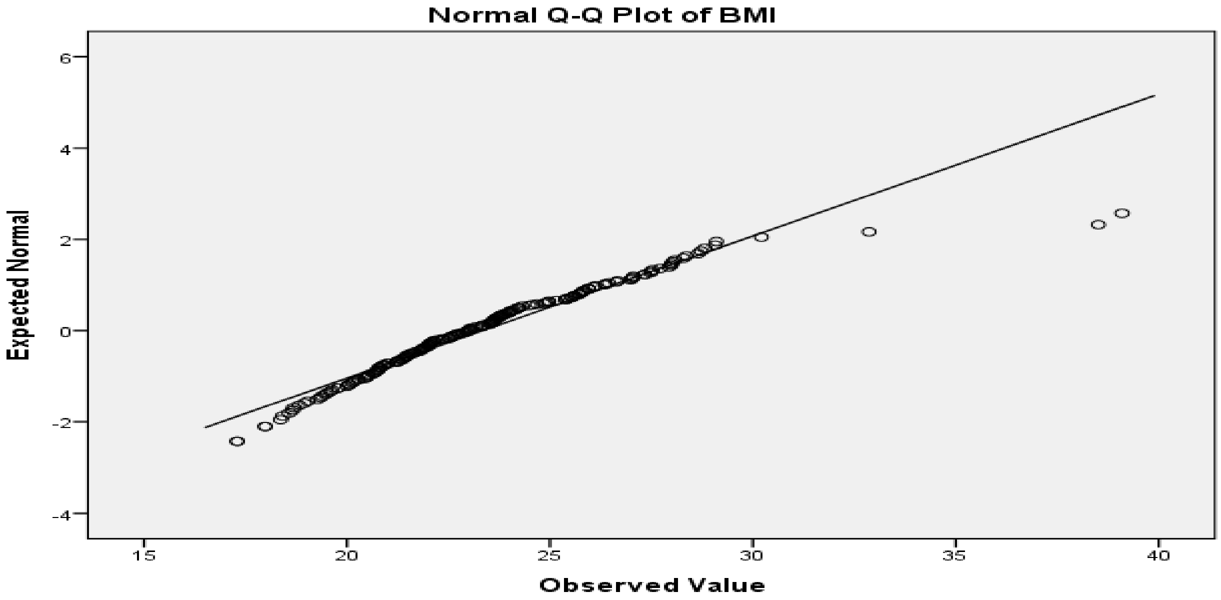

Comments: there exist outliers causing the distribution a bit of serious skewed to right. So may apply nonparametric method (Mann-Whitney test).

Remarks: There exist outliers in BMI, one way we can solved is remove these two outlier then redo the analysis. The other way we can transform using such as square root or log function to be more normal distribution like. The last option we can use the conservative approach of nonparametric method of Mann-Whitney test. For the safety we may use the last one. Also all normality test above shown the rejection of normality assumption.

After we applied the Mann-Whitney U test, we have

Test Statistics[a]

	age	BMI
Mann-Whitney U	3930.500	3845.500
Wilcoxon W	8880.500	7166.500
Z	-.227	-.472
Asymp. Sig. (2-tailed)	.820	.637

a. Grouping Variable: TCM2type

Remarks: These results may need to be replaced in Table1.

b) Retinal characteristics

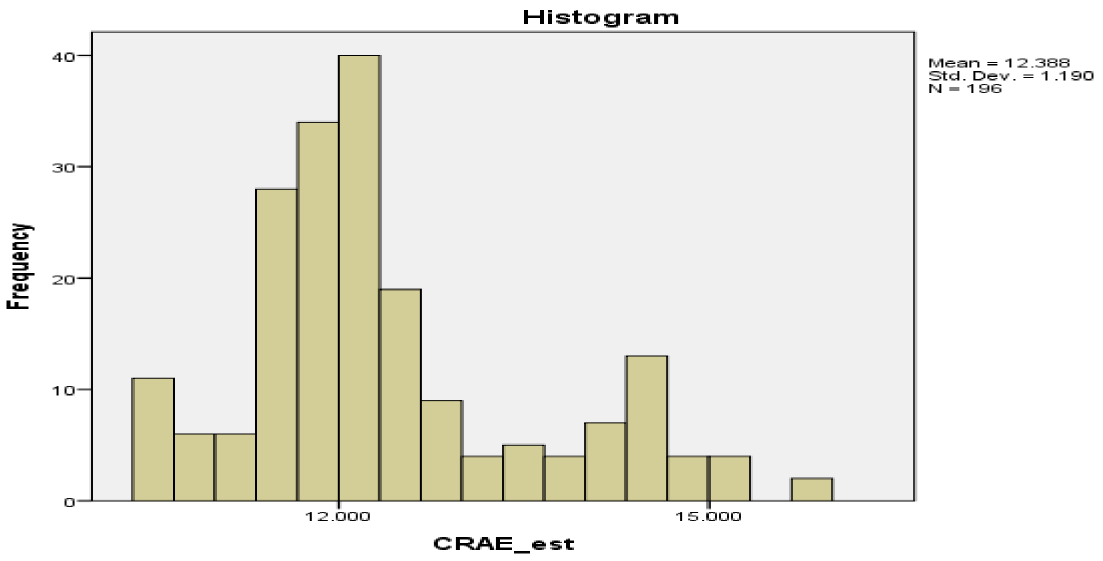

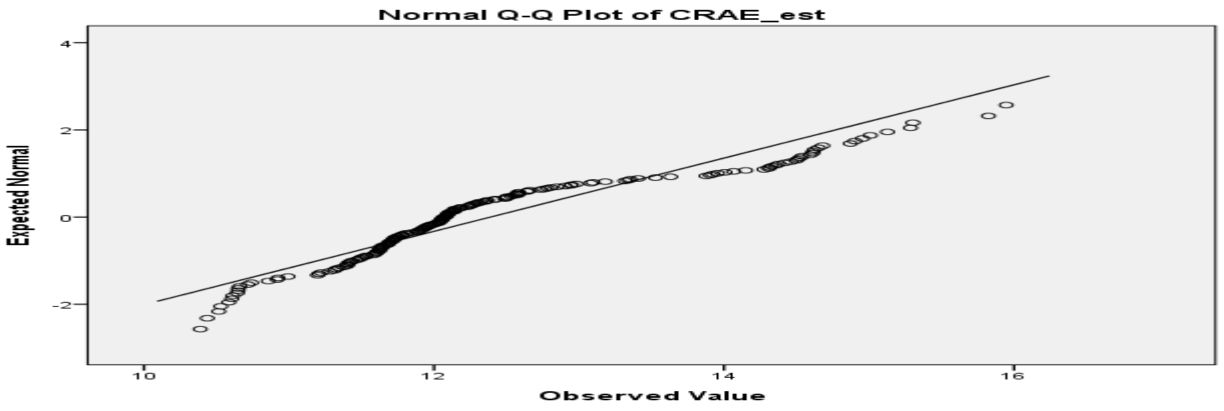

Comments: A bit of skewed to right

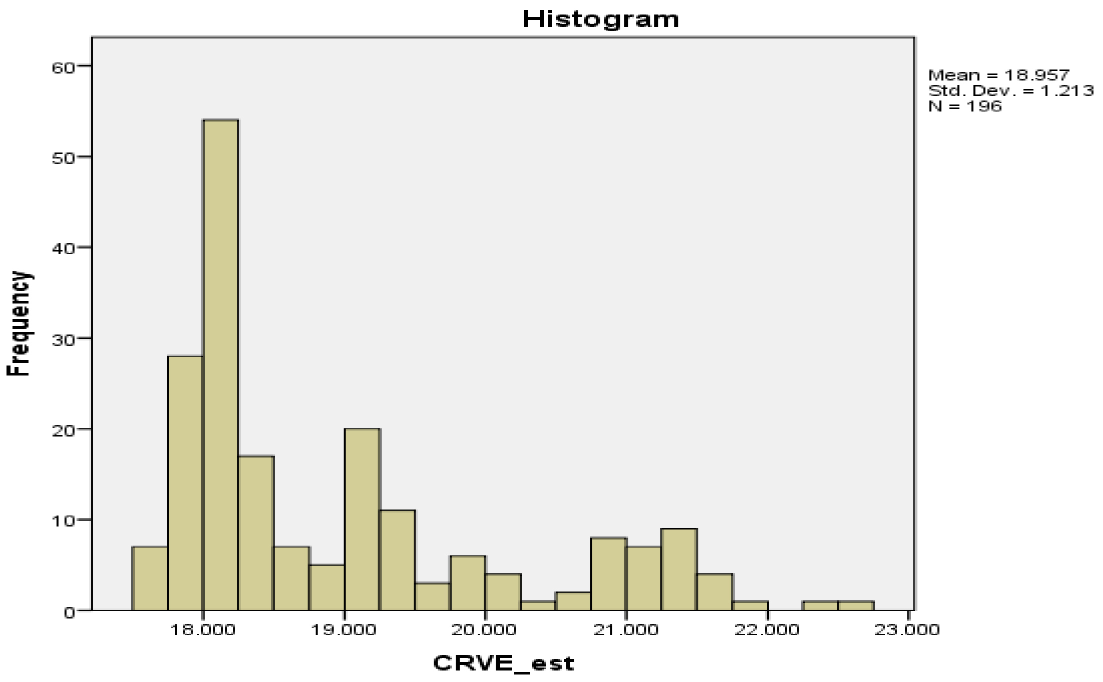

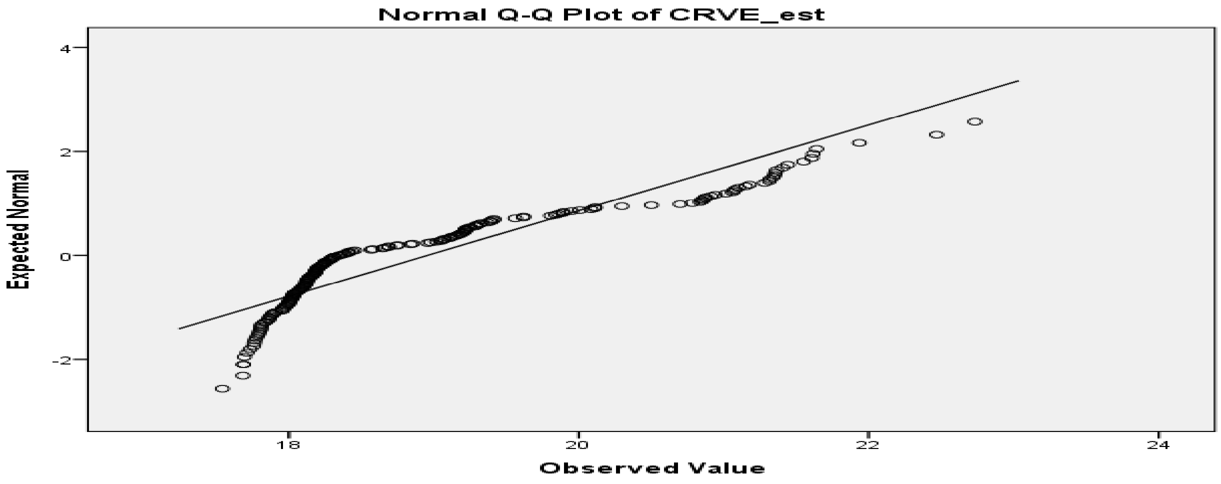

Comments: skewed to right

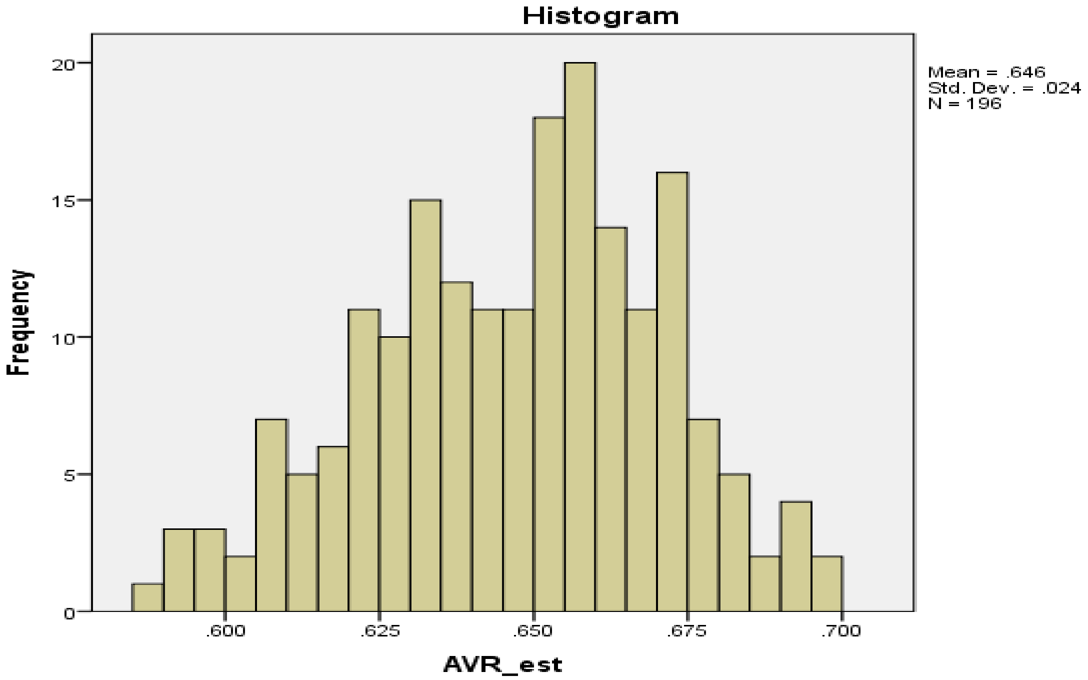

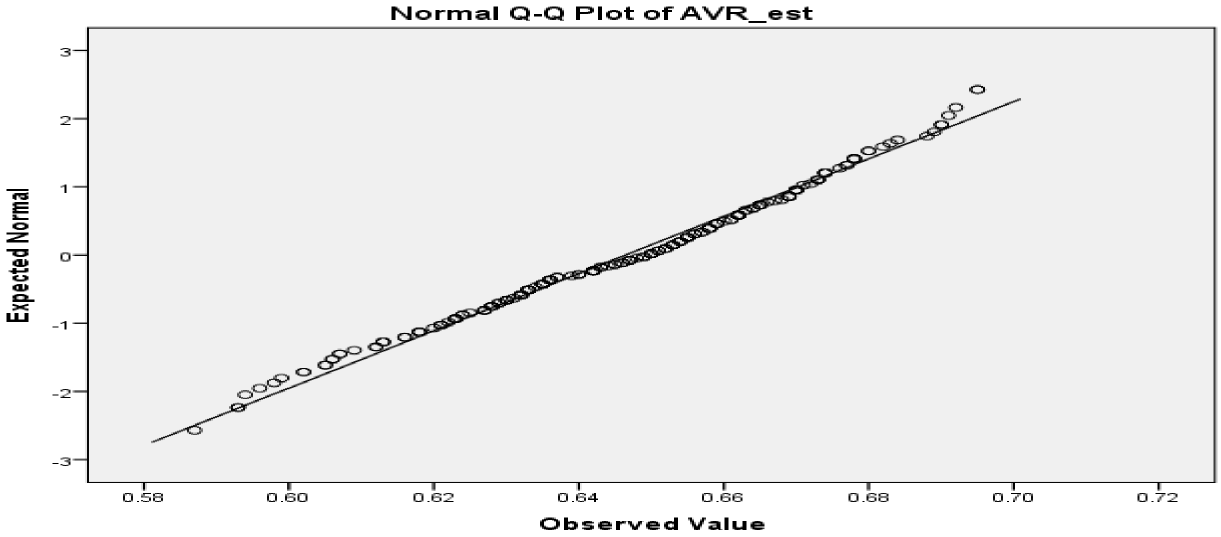

Comments: approximately normal

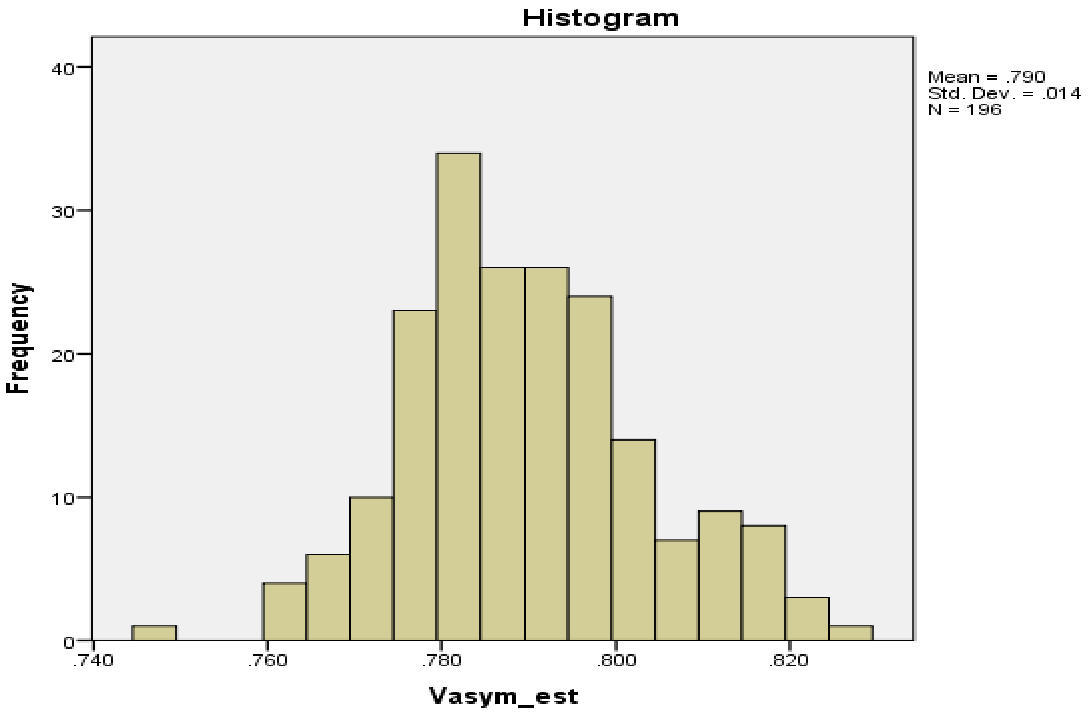

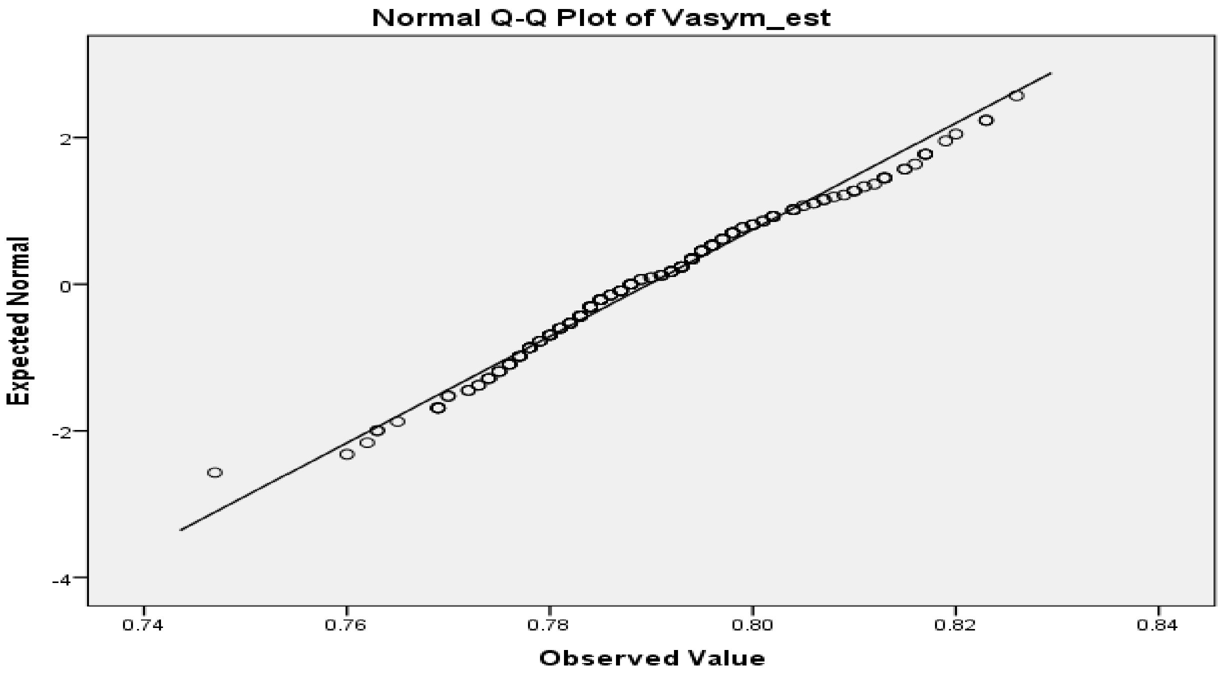

Comments: approximately normal.

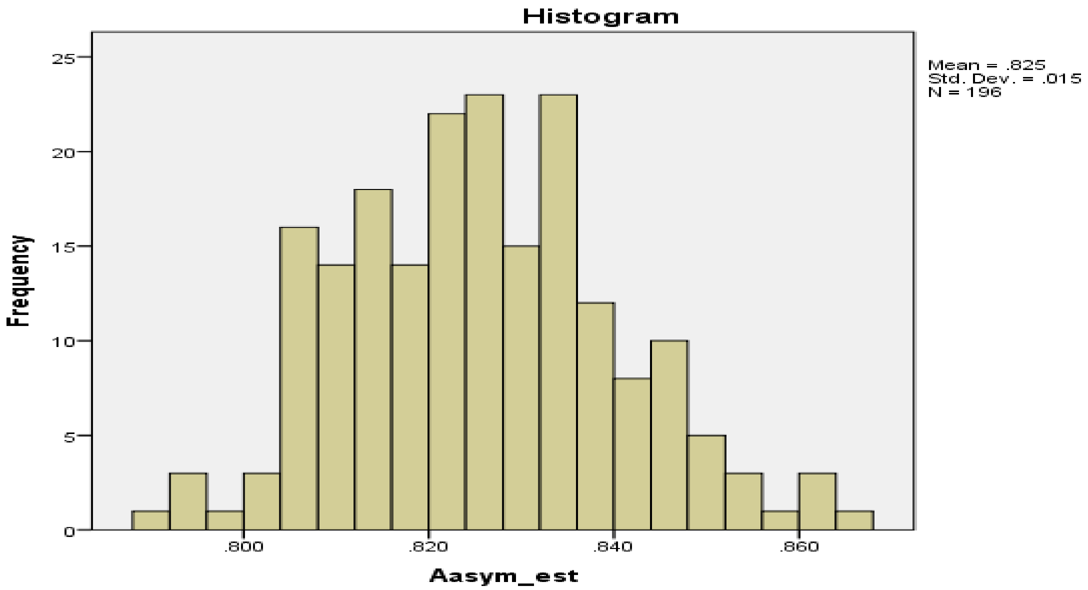
Histogram
Mean = .825
Std. Dev. = .015
N = 196
Frequency
25
20
15
10
5
0
.800
.820
.840
.860
Aasym_est

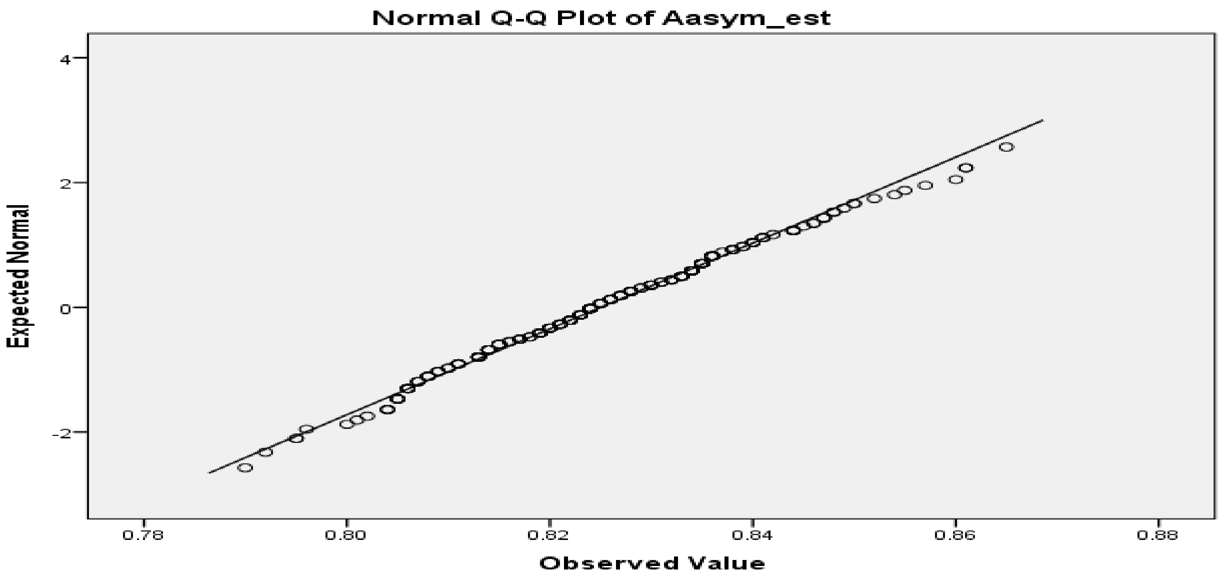
Normal Q-Q Plot of Aasym_est
Expected Normal
4
2
0
-2
0.78
0.80
0.82
0.84
0.86
0.88
Observed Value

Comments:

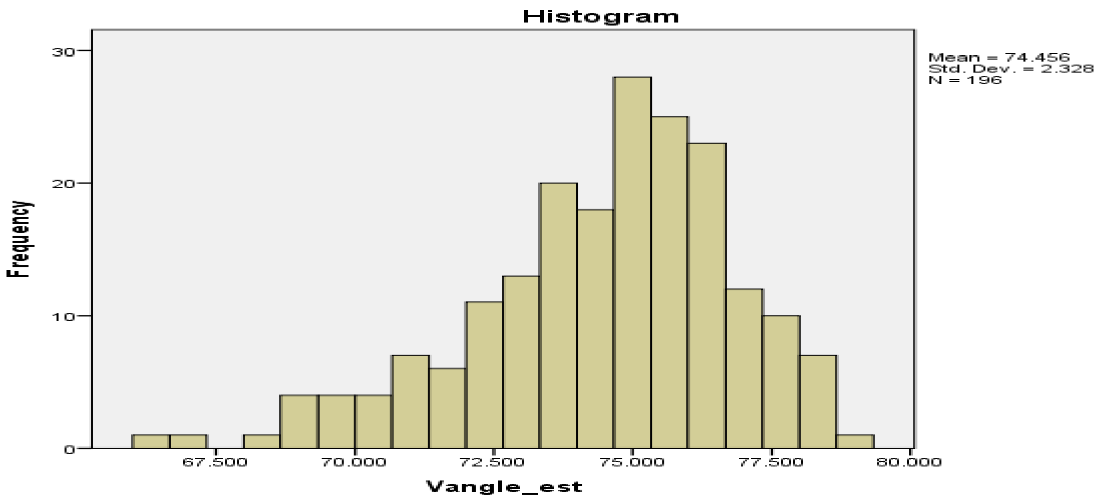

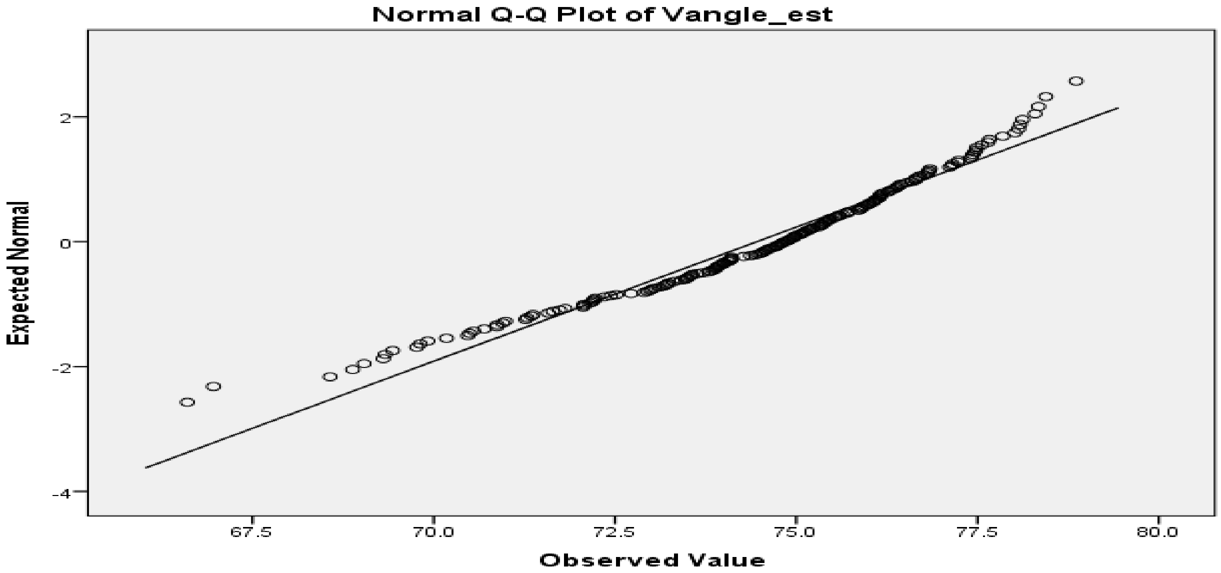

Comments: skewed to left.

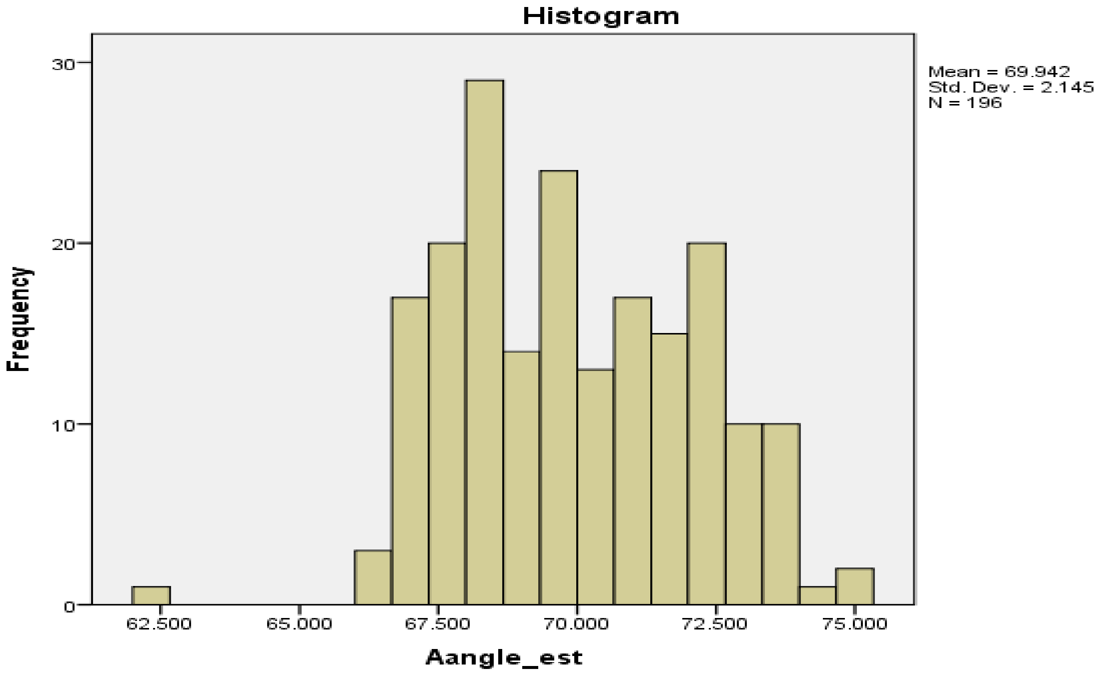

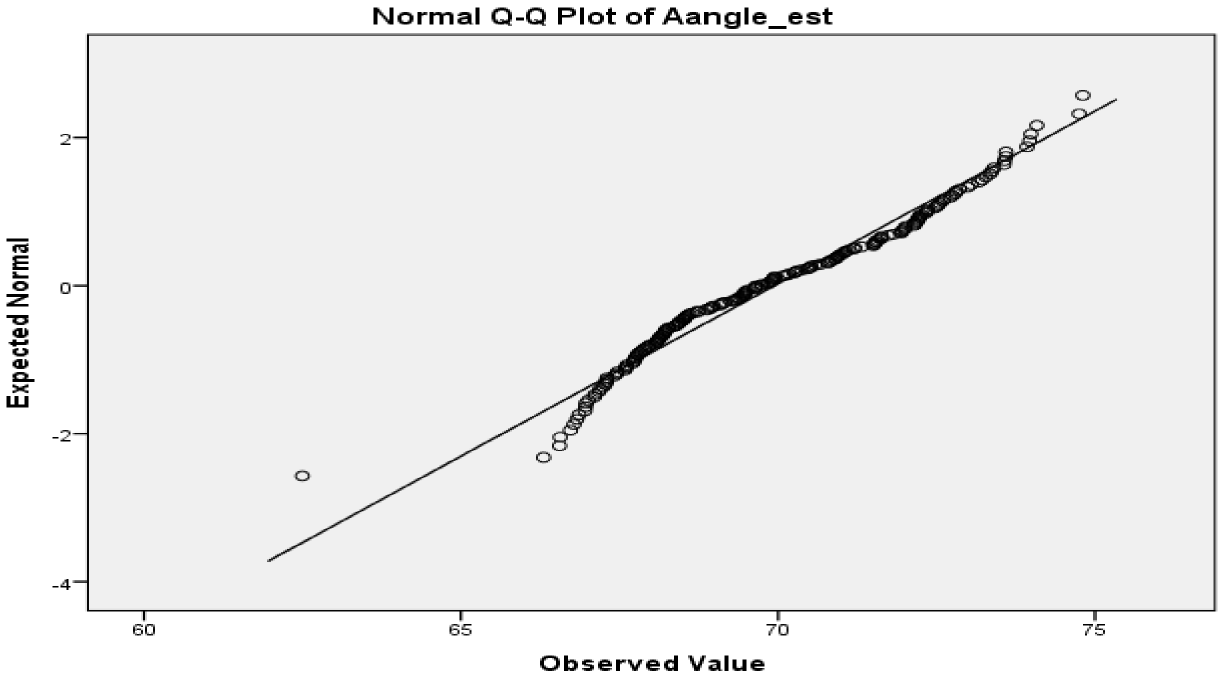

Comments: Thickness Biased in right

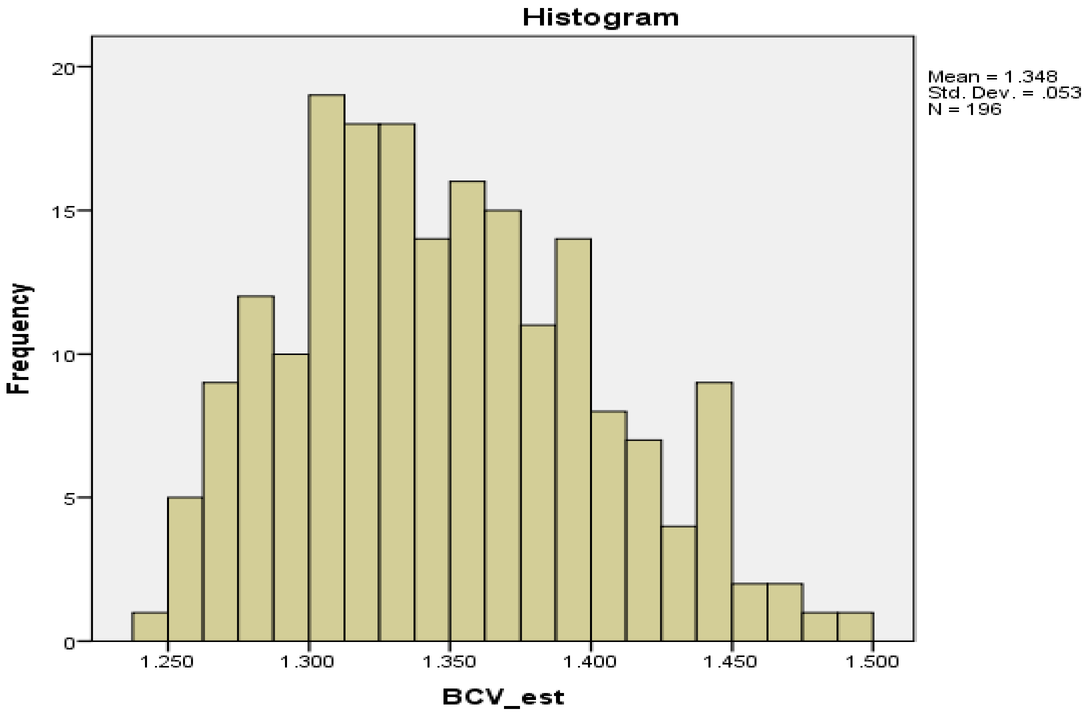

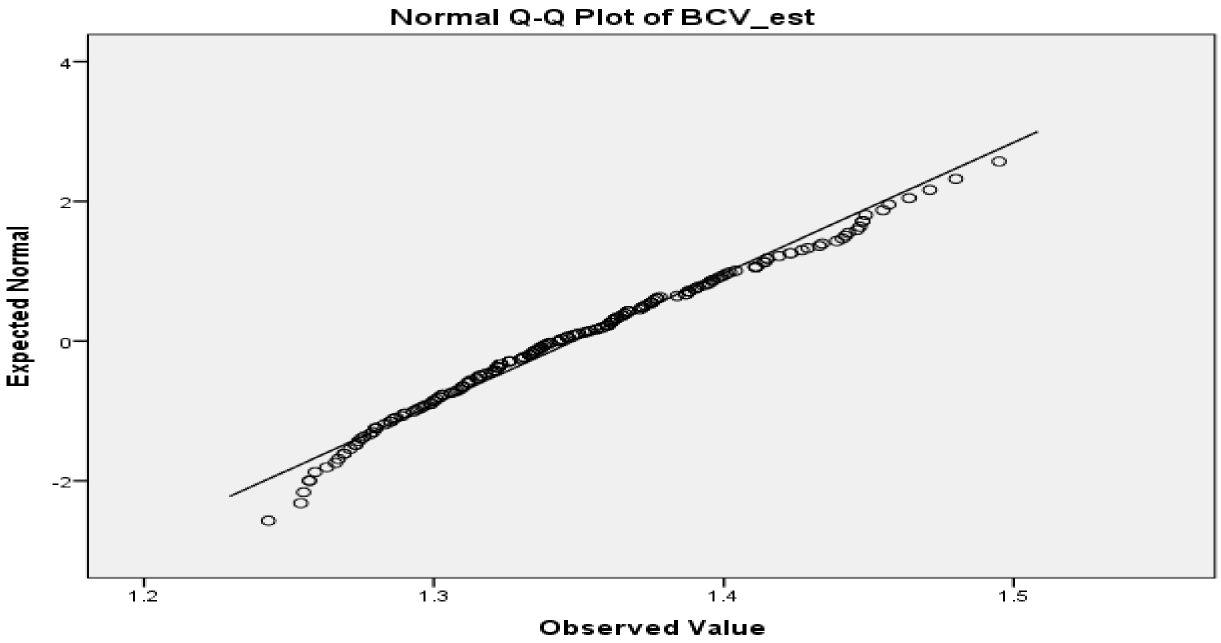

Comments: Thickness Biased in left

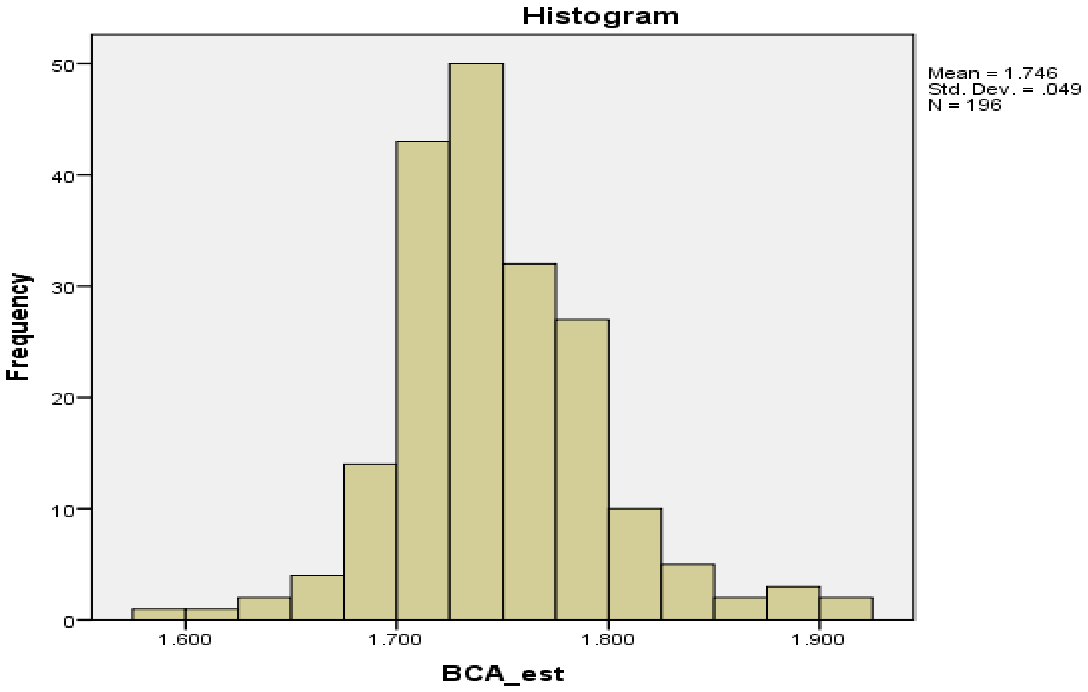

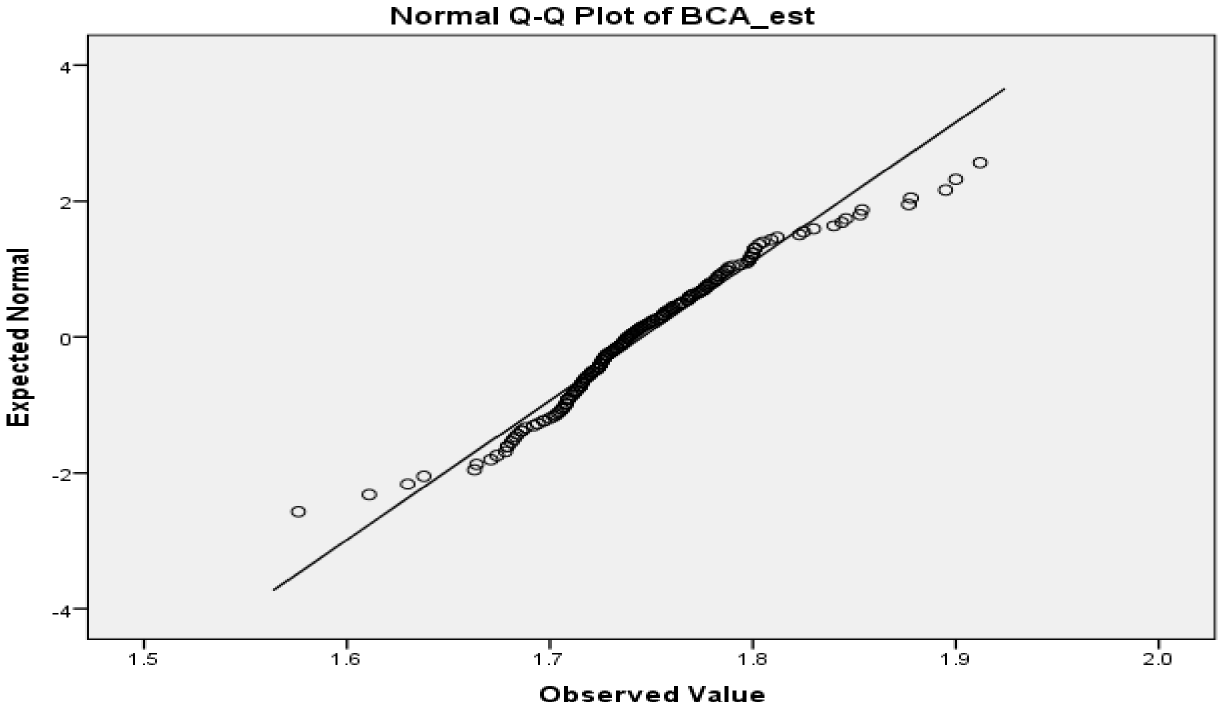

Comments: Thickness Biased in right (not in mormal)

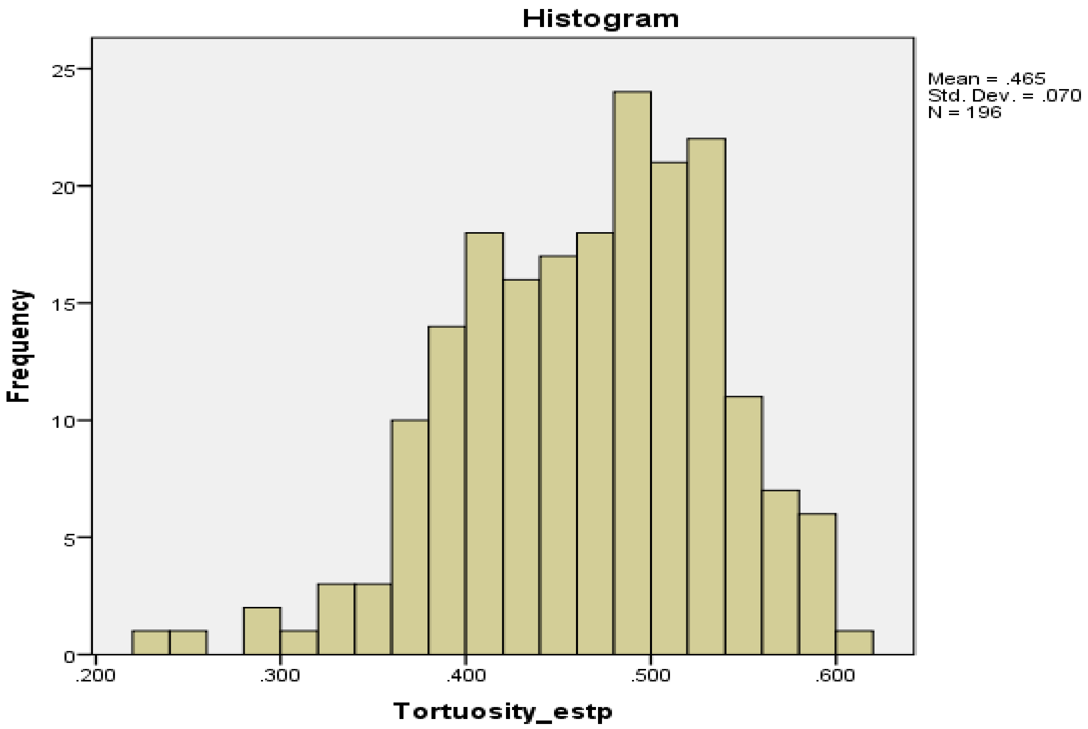

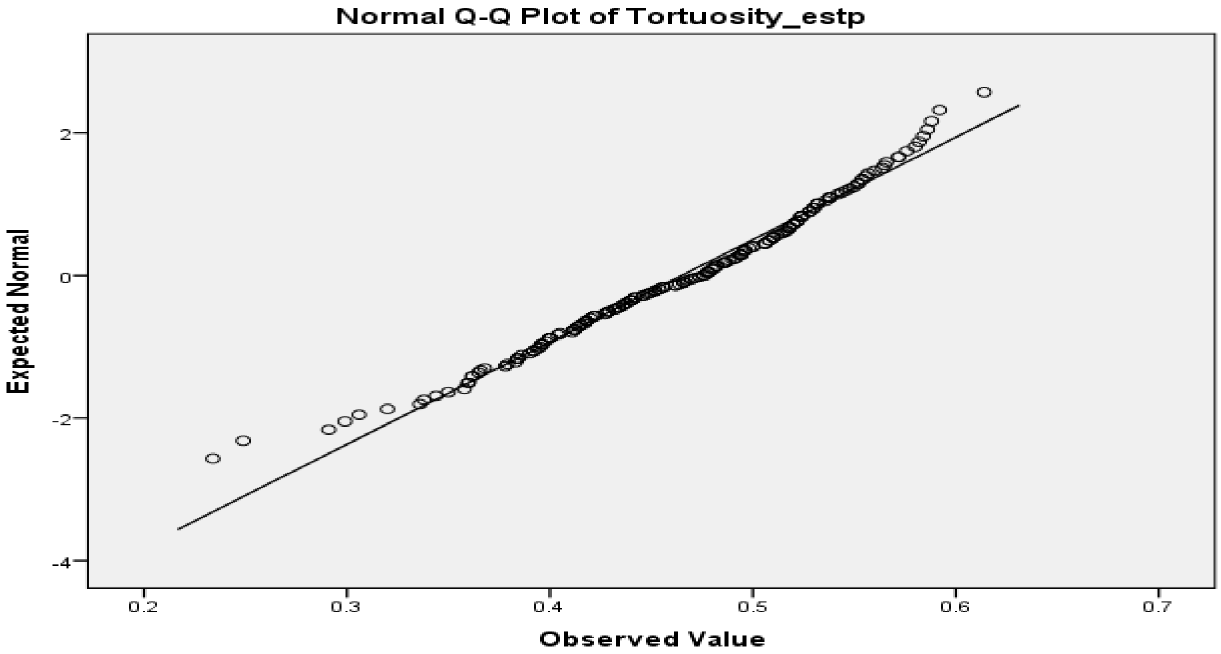

Comments: Thickness Biased in right (not in normal)

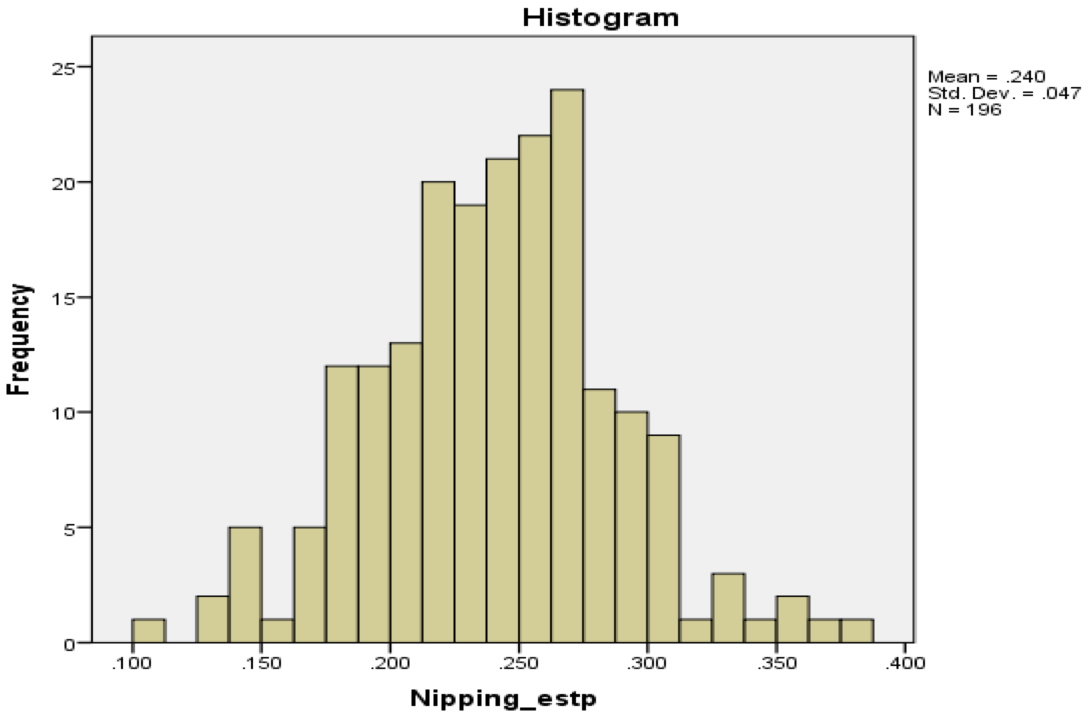

Comments: Thickness Biased in left

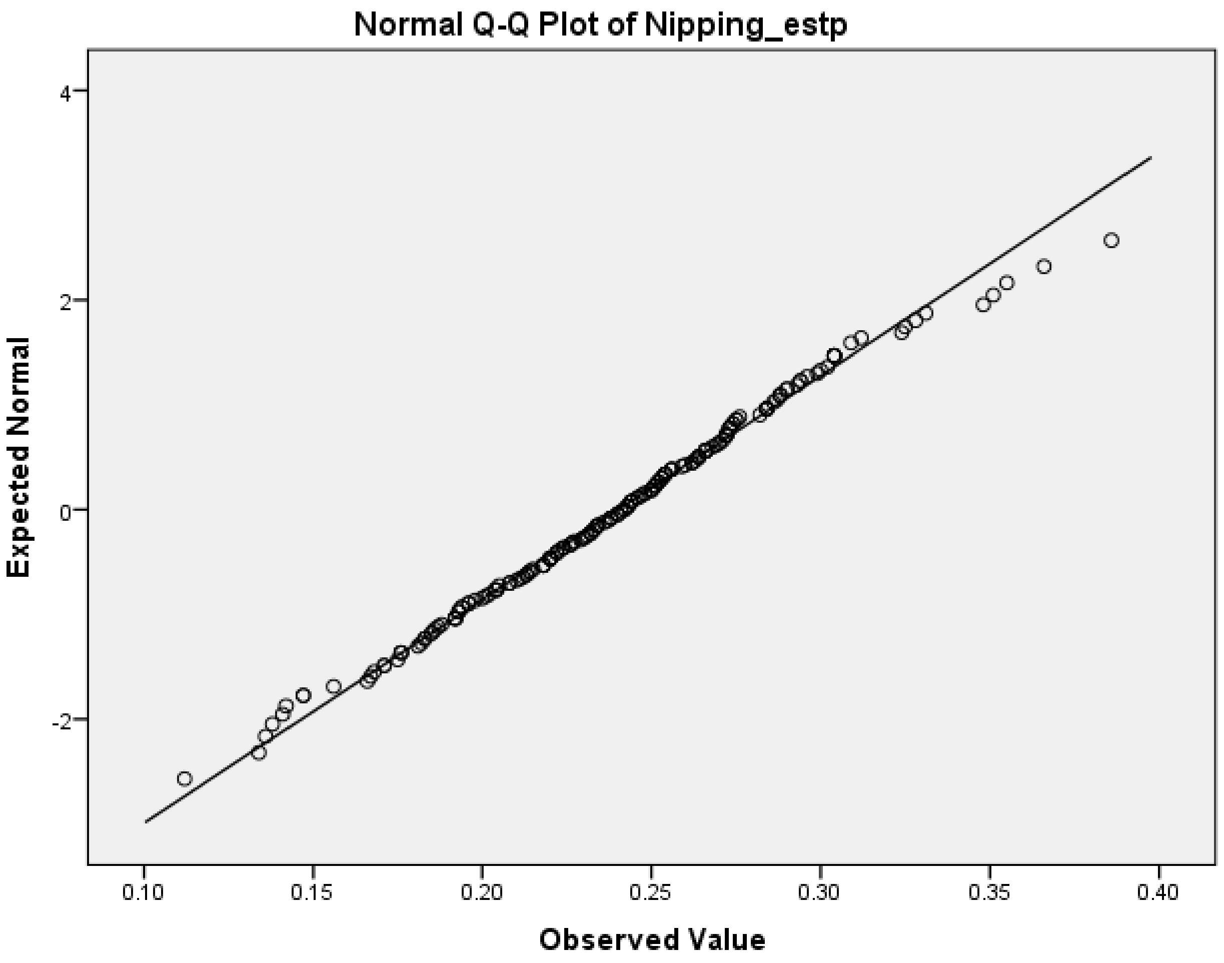

Normal Q-Q Plot of Nipping_estp
Expected Normal
Observed Value
4
2
0
-2
0.10
0.15
0.20
0.25
0.30
0.35
0.40

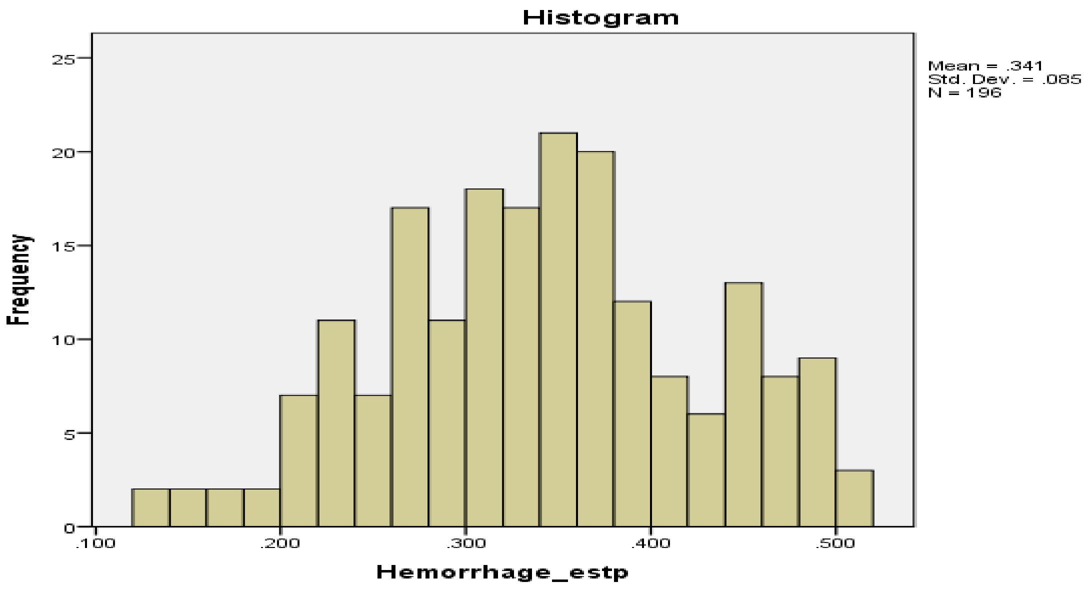

Histogram
Frequency
Hemorrhage_estp
Mean = .341
Std. Dev. = .085
N = 196
25
20
15
10
5
0
.100
.200
.300
.400
.500

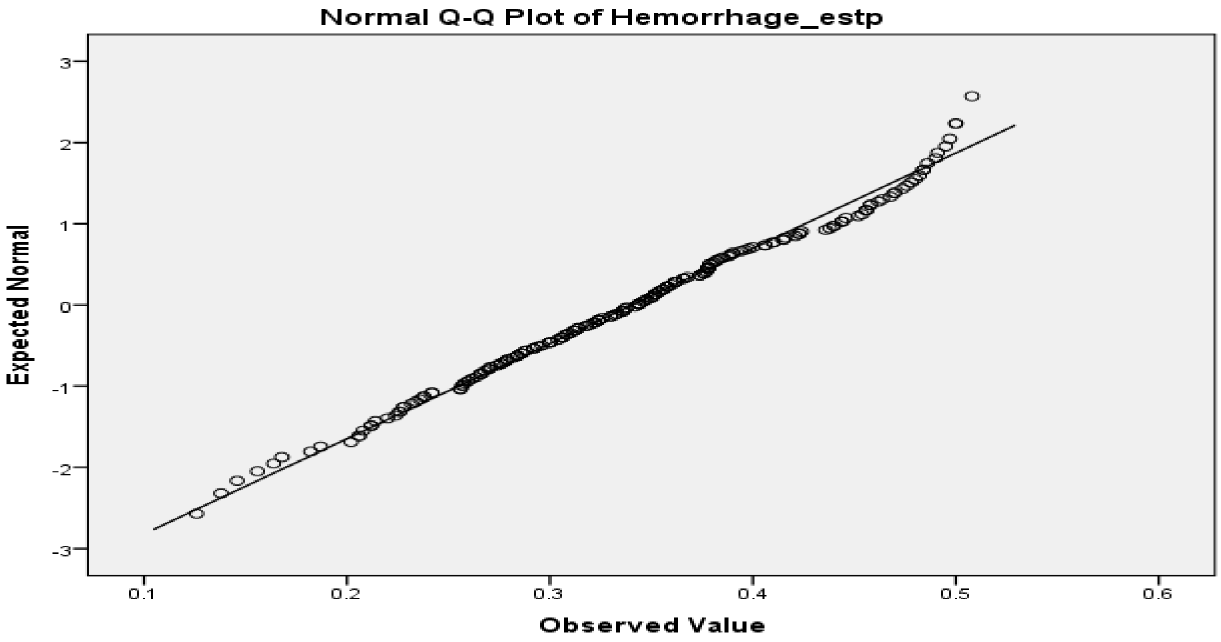

Comments: approximately normal distribution

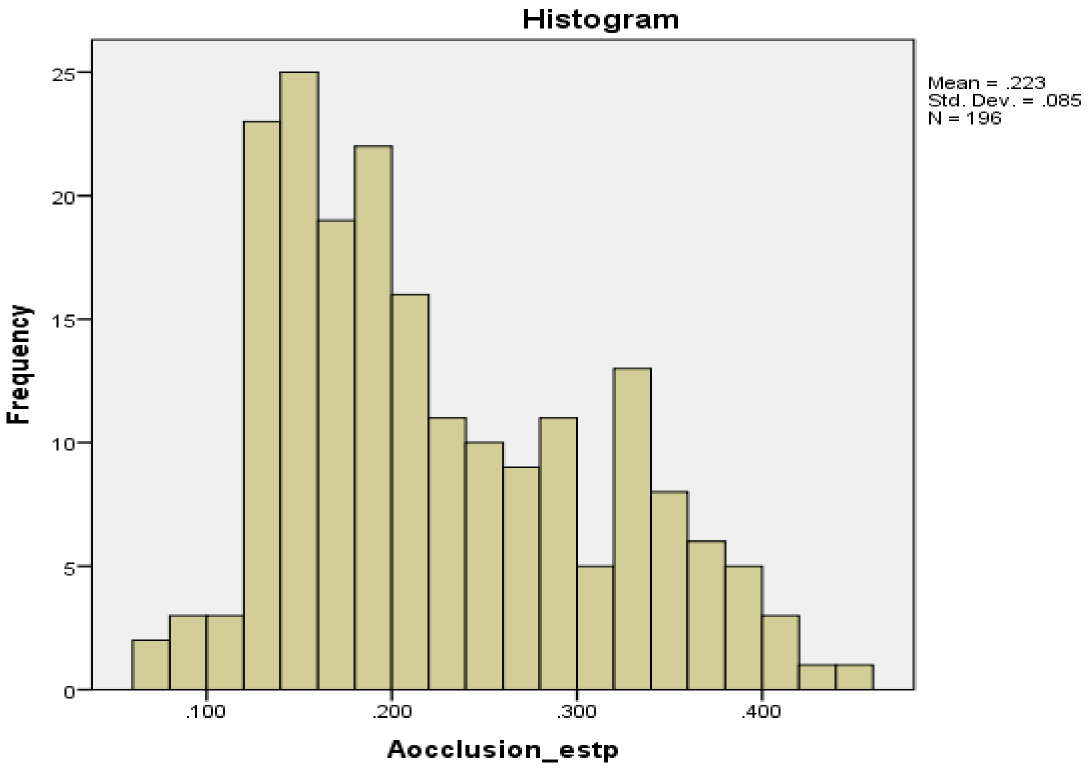

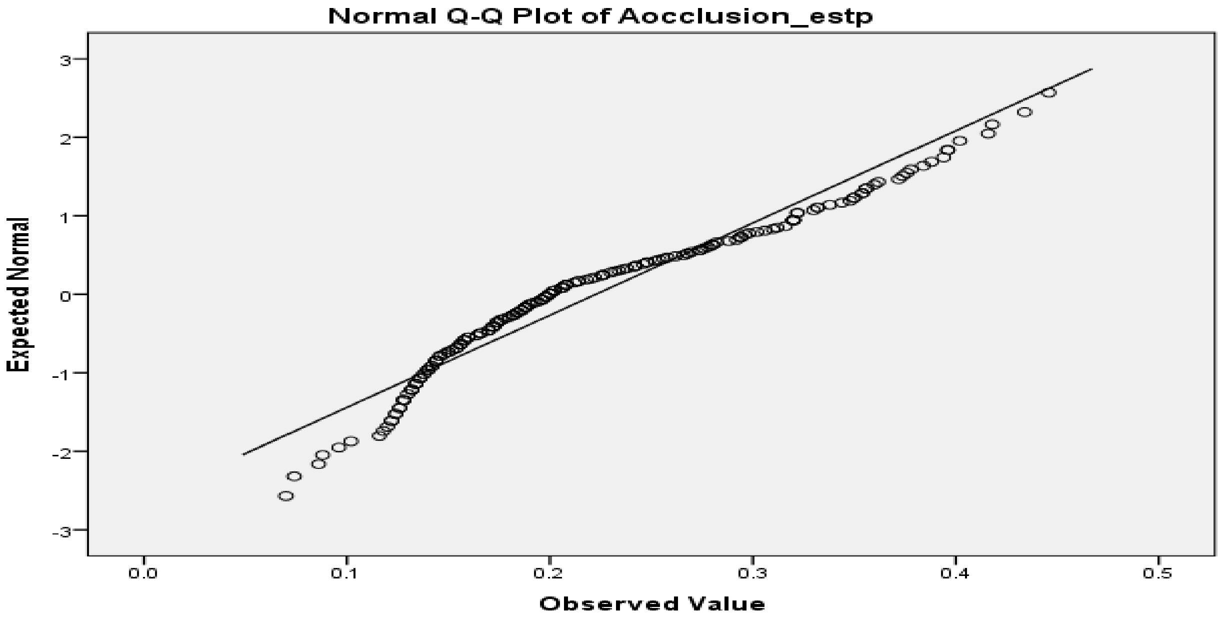

Comments: skewed to right

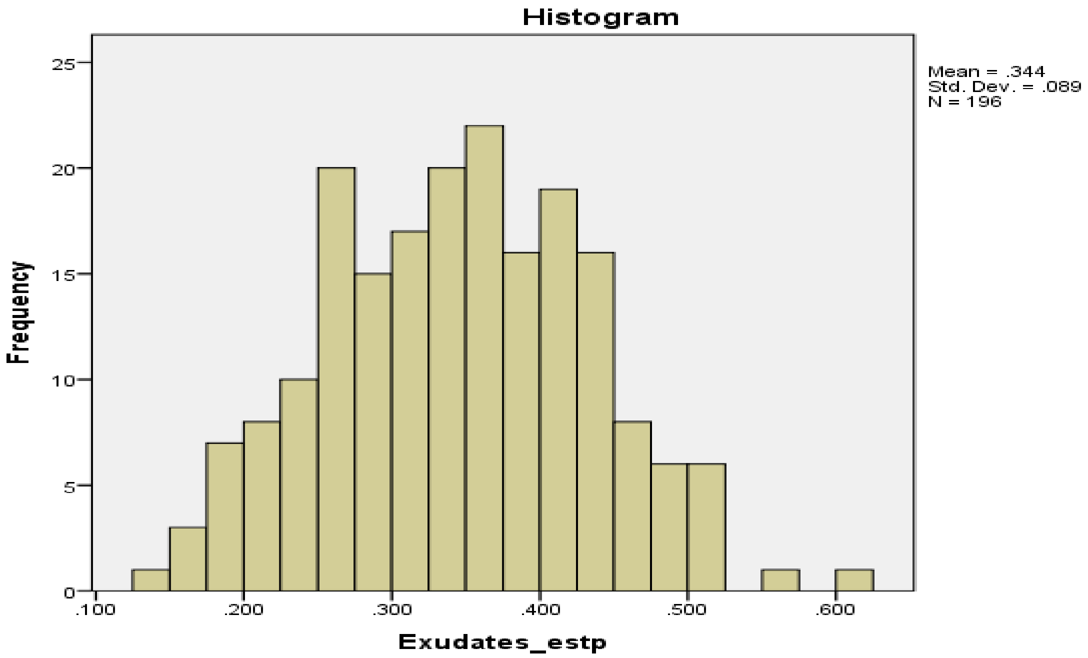

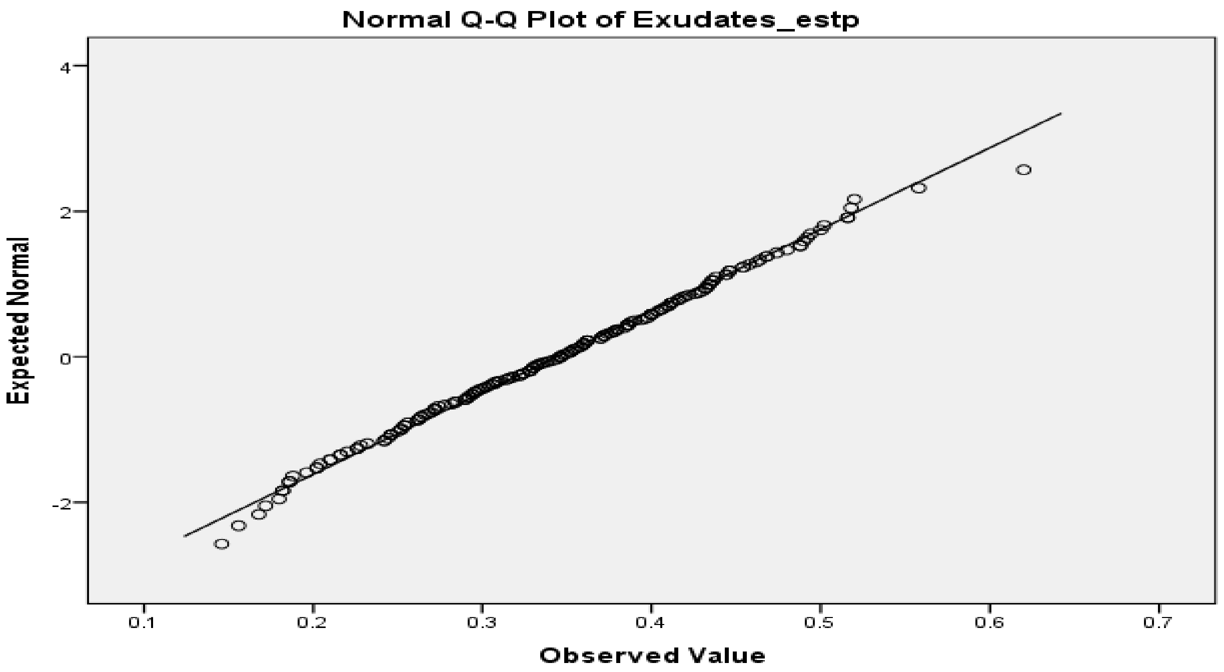

Comments: approximately normal distribution

Tests of Normality

	Kolmogorov-Smirnov[a]			Shapiro-Wilk		
	Statistic	df	Sig.	Statistic	df	Sig.
CRAE_est	.161	196	.000	.908	196	.000
CRVE_est	.204	196	.000	.842	196	.000
AVR_est	.065	196	.045	.986	196	.046
Vasym_est	.072	196	.016	.986	196	.055
Aasym_est	.043	196	.200*	.993	196	.426
Vangle_est	.085	196	.002	.961	196	.000
Aangle_est	.085	196	.002	.970	196	.000
BCV_est	.060	196	.088	.982	196	.012
BCA_est	.070	196	.019	.964	196	.000
Tortuosity_estp	.069	196	.026	.982	196	.011
Nipping_estp	.039	196	.200*	.995	196	.725
Hemorrhage_estp	.047	196	.200*	.987	196	.064
Aocclusion_estp	.125	196	.000	.944	196	.000
Exudates_estp	.035	196	.200*	.994	196	.608

*. This is a lower bound of the true significance.

a. Lilliefors Significance Correction

Remark:

Notice that the **Shapiro-Wilk test** is kind of a specific **test** for normality, whereas the method used by **Kolmogorov-Smirnov test** is more general, but less powerful (meaning it correctly rejects the null hypothesis of normality less often). Also there is such a test (equivalent to the Kolmogorov-Smirnov with estimated parameters) - the Lilliefors test; the normality-test version could be validly compared to the Shapiro-Wilk (and will generally have lower power).

After applied mann-whitney u test, we have

Test Statistics[a]

	CRAE_est	CRVE_est	AVR_est	Vangle_est	Aangle_est	BCV_est	BCA_est	Tortuosity_estp	Aocclusion_estp
Mann-Whitney U	3933.000	3693.500	3464.000	3709.500	3770.500	3780.500	3821.500	3239.000	3734.000
Wilcoxon W	7254.000	7014.500	8414.000	8659.500	8720.500	8730.500	7142.500	6560.000	8684.000
Z	-.220	-.909	-1.569	-.863	-.687	-.658	-.541	-2.216	-.792
Asymp. Sig. (2-tailed)	.826	.364	.117	.388	.492	.510	.589	.027	.428

a. Grouping Variable: TCM2type

Comments: above results may need to be replaced in Table1.

Part2. For categorical variables (only clinical characteristics)

Crosstab

Count

		TCM2type		Total
		0	1	
Gender	0	28	19	47
	1	53	80	133
Total		81	99	180

Chi-Square Tests

	Value	df	Asymp. Sig. (2-sided)	Exact Sig. (2-sided)	Exact Sig. (1-sided)
Pearson Chi-Square	5.459[a]	1	.019		
Continuity Correction[b]	4.691	1	.030		
Likelihood Ratio	5.451	1	.020		
Fisher's Exact Test				.026	.015
Linear-by-Linear Association	5.429	1	.020		
N of Valid Cases	180				

a. 0 cells (0.0%) have expected count less than 5. The minimum expected count is 21.15.

b. Computed only for a 2x2 table

Crosstab

Count

		TCM2type		Total
		0	1	
Drinking_History	0	70	74	144
	1	11	25	36
Total		81	99	180

Chi-Square Tests

	Value	df	Asymp. Sig. (2-sided)	Exact Sig. (2-sided)	Exact Sig. (1-sided)
Pearson Chi-Square	3.793[a]	1	.051		
Continuity Correction[b]	3.099	1	.078		
Likelihood Ratio	3.899	1	.048		
Fisher's Exact Test				.062	.038
Linear-by-Linear Association	3.772	1	.052		
N of Valid Cases	180				

a. 0 cells (0.0%) have expected count less than 5. The minimum expected count is 16.20.

b. Computed only for a 2x2 table

Crosstab

Count

		TCM2type		Total
		0	1	
insomnia	0	44	70	114
	1	37	29	66
Total		81	99	180

Chi-Square Tests

	Value	df	Asymp. Sig. (2-sided)	Exact Sig. (2-sided)	Exact Sig. (1-sided)
Pearson Chi-Square	5.151[a]	1	.023		
Continuity Correction[b]	4.470	1	.035		
Likelihood Ratio	5.151	1	.023		
Fisher's Exact Test				.029	.017
Linear-by-Linear Association	5.122	1	.024		
N of Valid Cases	180				

a. 0 cells (0.0%) have expected count less than 5. The minimum expected count is 29.70.

b. Computed only for a 2x2 table

Crosstab

Count

		TCM2type		Total
		0	1	
hypertension	0	19	22	41
	1	62	77	139
Total		81	99	180

Chi-Square Tests

	Value	df	Asymp. Sig. (2-sided)	Exact Sig. (2-sided)	Exact Sig. (1-sided)
Pearson Chi-Square	.039[a]	1	.844		
Continuity Correction[b]	.000	1	.986		
Likelihood Ratio	.039	1	.844		
Fisher's Exact Test				.860	.492
Linear-by-Linear Association	.038	1	.845		
N of Valid Cases	180				

a. 0 cells (0.0%) have expected count less than 5. The minimum expected count is 18.45.

b. Computed only for a 2x2 table

Crosstab

Count

		TCM2type		Total
		0	1	
diabetes	0	42	55	97
	1	39	44	83
Total		81	99	180

Chi-Square Tests

	Value	df	Asymp. Sig. (2-sided)	Exact Sig. (2-sided)	Exact Sig. (1-sided)
Pearson Chi-Square	.246[a]	1	.620		
Continuity Correction[b]	.119	1	.730		
Likelihood Ratio	.246	1	.620		
Fisher's Exact Test				.654	.365
Linear-by-Linear Association	.245	1	.621		
N of Valid Cases	180				

a. 0 cells (0.0%) have expected count less than 5. The minimum expected count is 37.35.

b. Computed only for a 2x2 table

Crosstab

Count

		TCM2type		Total
		0	1	
Coronary_Heart_Disease	0	61	71	132
	1	20	28	48
Total		81	99	180

Chi-Square Tests

	Value	df	Asymp. Sig. (2-sided)	Exact Sig. (2-sided)	Exact Sig. (1-sided)
Pearson Chi-Square	.294[a]	1	.588		
Continuity Correction[b]	.139	1	.709		
Likelihood Ratio	.295	1	.587		
Fisher's Exact Test				.615	.356
Linear-by-Linear Association	.292	1	.589		
N of Valid Cases	180				

a. 0 cells (0.0%) have expected count less than 5. The minimum expected count is 21.60.

b. Computed only for a 2x2 table

Crosstab

Count

		TCM2type		Total
		0	1	
Abnormal_lipid_metabolism	0	54	70	124
	1	27	29	56
Total		81	99	180

Chi-Square Tests

	Value	df	Asymp. Sig. (2-sided)	Exact Sig. (2-sided)	Exact Sig. (1-sided)
Pearson Chi-Square	.339[a]	1	.560		
Continuity Correction[b]	.177	1	.674		
Likelihood Ratio	.339	1	.561		
Fisher's Exact Test				.628	.336
Linear-by-Linear Association	.337	1	.561		
N of Valid Cases	180				

a. 0 cells (0.0%) have expected count less than 5. The minimum expected count is 25.20.

b. Computed only for a 2x2 table

Crosstab

Count

	TCM2type	Total

		0	1	
High_homocysteine	0	74	86	160
	1	7	13	20
Total		81	99	180

Chi-Square Tests

	Value	df	Asymp. Sig. (2-sided)	Exact Sig. (2-sided)	Exact Sig. (1-sided)
Pearson Chi-Square	.909[a]	1	.340		
Continuity Correction[b]	.511	1	.475		
Likelihood Ratio	.926	1	.336		
Fisher's Exact Test				.475	.239
Linear-by-Linear Association	.904	1	.342		
N of Valid Cases	180				

a. 0 cells (0.0%) have expected count less than 5. The minimum expected count is 9.00.

b. Computed only for a 2x2 table

Comments: Based on above results, we use the general chi-square test (i.e., Person Chi-square test) except the last variable we may apply the test of continuity correction since the minimum expected count is between 5 to10. (No need Fisher's Exact Test at all here).
