# Supplementary material for: The Discriminative Efficacy of Retinal Characteristics on Two Traditional Chinese Syndromes in Association with Ischemic Stroke
Source: Evid Based Complement Alternat Med. 2020 Mar 26;2020:6051831. doi: 10.1155/2020/6051831 (PMC7136789; doi:10.1155/2020/6051831)
Supplement: Supplementary Materials — These are the retinal images of 196 patients recruited in the study. Supplementary for univariate analysis. [file 6051831.f1.zip › 6051831.f1/Retinal images.pdf]

These are some retinal images of the 196 patients recruited between 2014 and 2018 in the department of acupuncture and moxibustion of Shenzhen traditional Chinese Medicine Hospital. All these pictures were taken on the 2nd day of hospital admission using the SmartScope Ey4 Camera (Optomed, Finland). To ensure the compatibility of the parameters, all the retinal images were scaled to 1365*1024 pixels and saved in the .jpg format.

PS： The patient has the ownership of the photo and cannot reprint them without permission

I Partial fundus photos of 80 patients with Qi deficiency and blood stasis syndrome

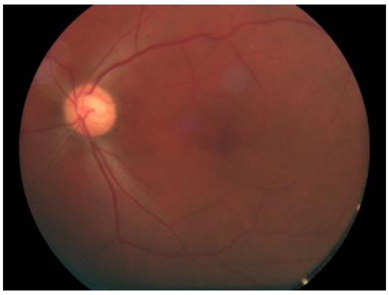

Q-PIC 001

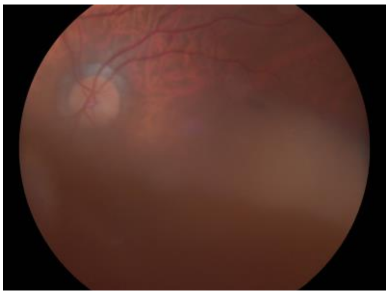

Q-PIC 002

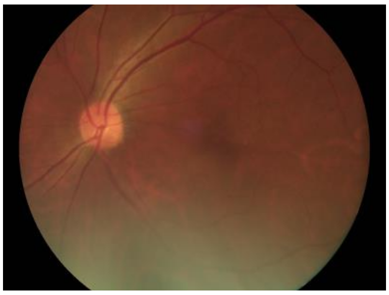

Q-PIC 003

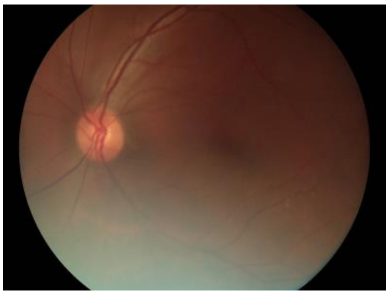

Q-PIC 004

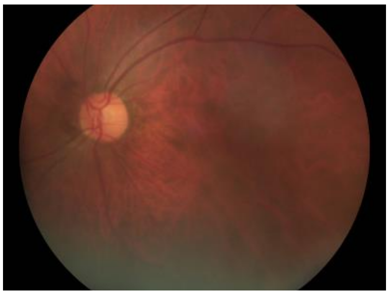

Q-PIC 005

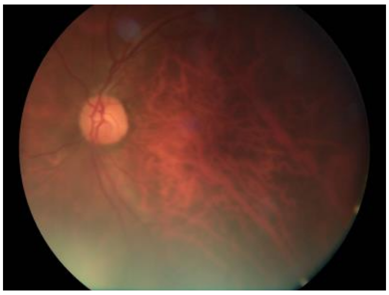

Q-PIC 006

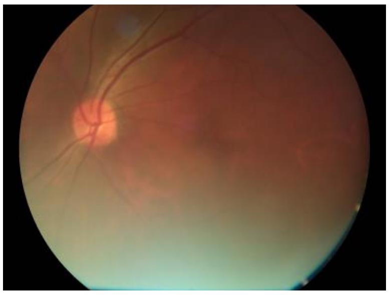

Q-PIC 007

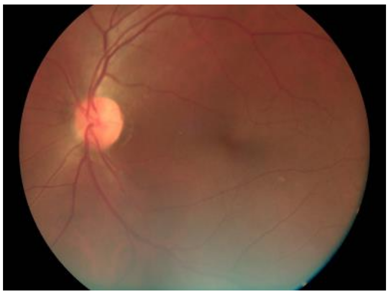

Q-PIC 008

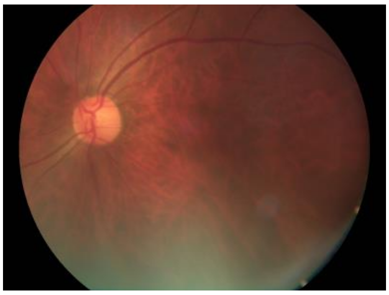

Q-PIC 009

II Partial fundus photos of 100 patients with phlegm stasis in channels syndrome

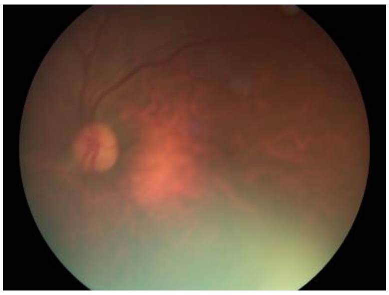

P-PIC 001

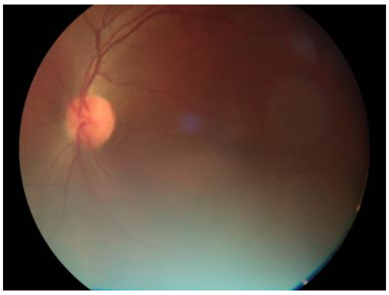

P-PIC 002

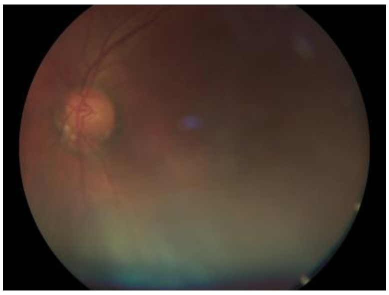

P-PIC 003

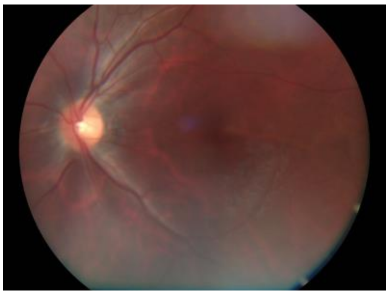

P-PIC 004

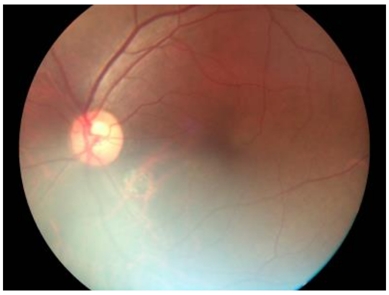

P-PIC 005

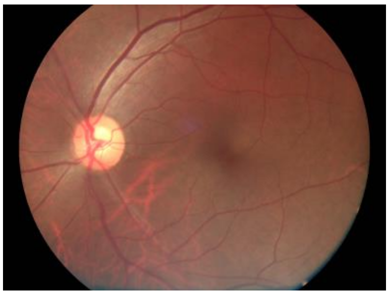

P-PIC 006

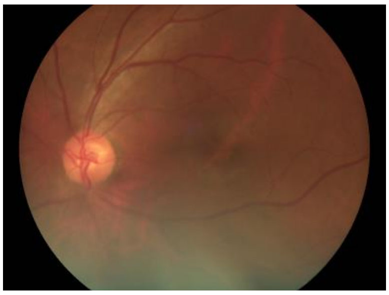

P-PIC 007

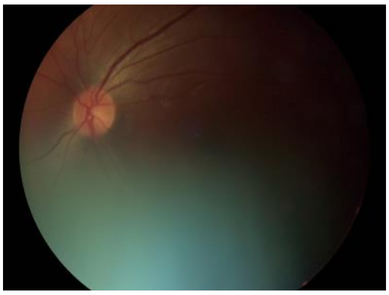

P-PIC 008

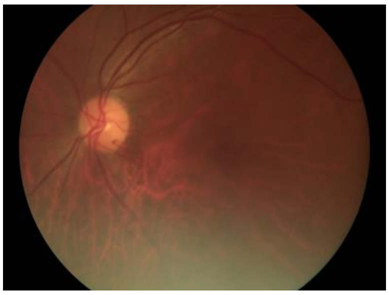

P-PIC 009

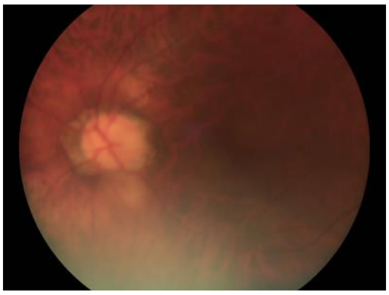

P-PIC 010

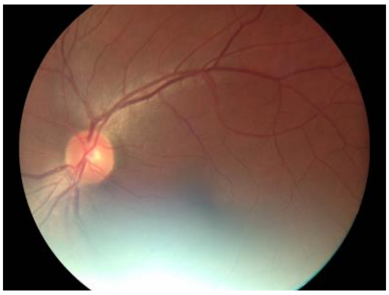

P-PIC 011

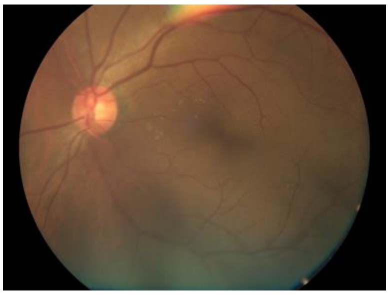

P-PIC 012

III Partial fundus photos of 16 patients with other syndrome

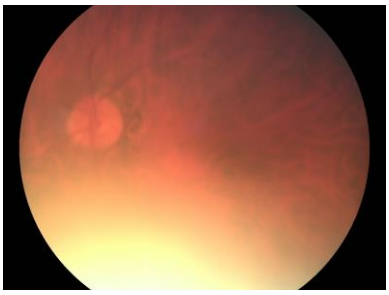

O-PIC 001

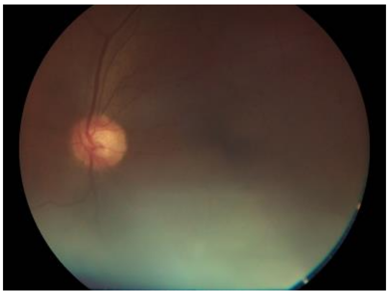

O-PIC 002

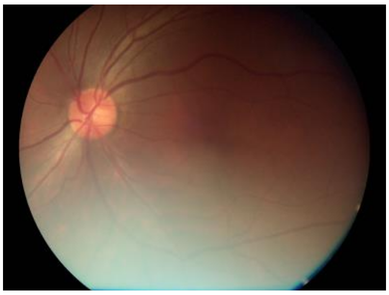

O-PIC 003

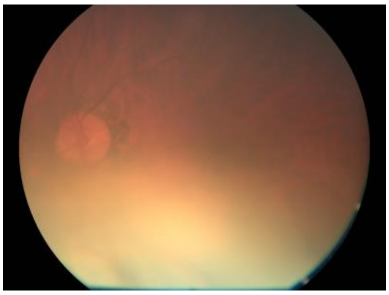

O-PIC 004

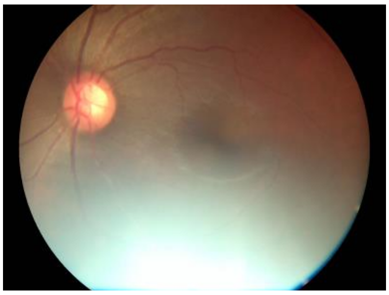

O-PIC 005

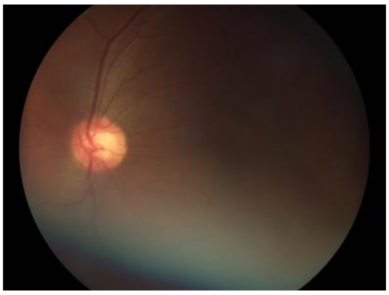

O-PIC 006

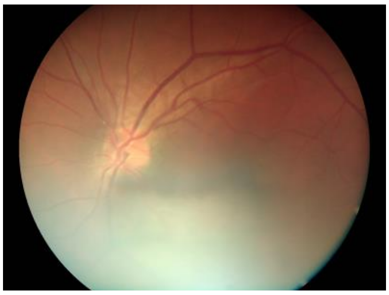

O-PIC 007

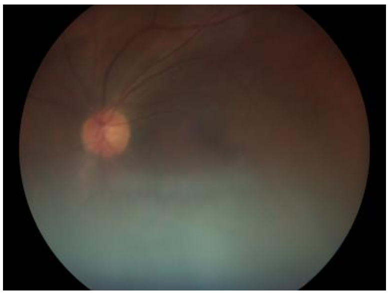

O-PIC 008

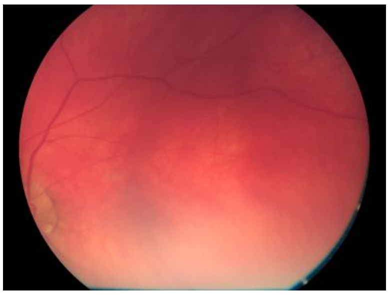

O-PIC 009

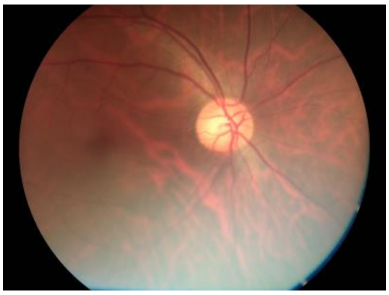

O-PIC 010

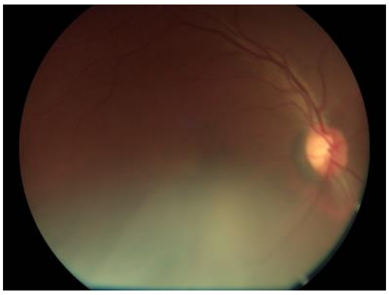

O-PIC 011

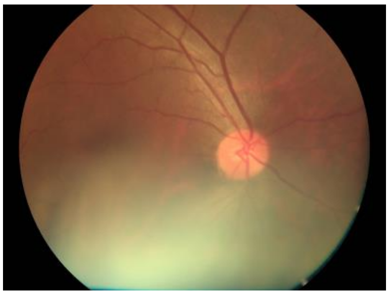

O-PIC 012
